# Bribe and Punishment: An Evolutionary Game-Theoretic Analysis of Bribery

**DOI:** 10.1371/journal.pone.0133441

**Published:** 2015-07-23

**Authors:** Prateek Verma, Supratim Sengupta

**Affiliations:** Department of Physical Sciences, Indian Institute of Science Education and Research Kolkata, Kolkata, India; Wenzhou University, CHINA

## Abstract

Harassment bribes, paid by citizens to corrupt officers for services the former are legally entitled to, constitute one of the most widespread forms of corruption in many countries. Nation states have adopted different policies to address this form of corruption. While some countries make both the bribe giver and the bribe taker equally liable for the crime, others impose a larger penalty on corrupt officers. We examine the consequences of asymmetric and symmetric penalties by developing deterministic and stochastic evolutionary game-theoretic models of bribery. We find that the asymmetric penalty scheme can lead to a reduction in incidents of bribery. However, the extent of reduction depends on how the players update their strategies over time. If the interacting members change their strategies with a probability proportional to the payoff of the alternative strategy option, the reduction in incidents of bribery is less pronounced. Our results indicate that changing from a symmetric to an asymmetric penalty scheme may not suffice in achieving significant reductions in incidents of harassment bribery.

## Introduction

Corruption is a pervasive problem in all nation states but especially in developing countries [1] where it leads to huge losses in revenue for the exchequer, erosion of social justice, violation of human rights and exploitation of vulnerable people in society. A recent EU report [2] on corruption in Europe estimated that corruption costs the European economy 120 billion euros annually. While corruption manifests itself in many forms, a particularly widespread and pernicious variety involves bribery. Bribery leads to substantial loss of revenue and also undermines democracy by undermining people’s faith in public institutions. A website (http://www.ipaidabribe.com/) set up by the non-governmental organization (NGO) Janaagraha for tracking bribery across India on the basis of voluntary disclosures of citizens reveal that more than 2.4 billion Indian Rupees (~ US$39 million) have been paid in bribes to corrupt officials since August, 2010. Bribery can be categorized into two classes; one where an individual or group pays a bribe to illegally get access to a product or service (collusive bribes) and another class where an individual or a group has to pay a bribe to get a service that they are legally entitled to as citizens or residents. Examples of the latter class of bribes include bribes given to register a new property, obtain a passport, driver’s license, tax refund, a new electricity connection etc. This class of bribes were termed *harassment bribes* by Basu (2011). He argued that bribery incidents of this kind would be greatly reduced if the bribe-giver was not penalized when caught [3], in contravention of the current law where the bribe taker and bribe giver are equally liable for the crime and hence punishable to the same degree. According to Basu (2011) this asymmetry in punishing only the bribe-taker but not the bribe-giver would encourage bribe-givers to report incidents of bribery thereby increasing the likelihood of bribe-takers being prosecuted for their crime. Over time, this would eventually lead to reduction of bribery incidents by discouraging bribe-takers from demanding bribes.

While symmetric punishment is enshrined in the criminal laws of several countries like India, U.S.A., UK, France; laws of countries like China, Japan and Russia require imposing a greater penalty on bribe-takers relative to bribe-givers [4]. However, the question of which policy is more effective in reducing incidents of bribery, remains a controversial one [5] and therefore ripe for further analysis using a variety of techniques.

Abbink and collaborators initiated [6] the experimental study of bribery and extensively analysed [7] (see also [8,9]]) various aspects of bribery using laboratory experiments. Based on the analysis of such experiments, various policy measures [10–12] have also been suggested to reduce corruption. The effects of framing on the tendency to offer bribes [13,14] as well as the effect of cultural influences [15–17] on the behaviour of the principal players in a bribery game have also been investigated. However, such analysis was confined to collusive bribes in which bribes are given to illegally gain special favours. Since Basu put forward his proposal, a few experimental [18,19] and theoretical (Dufwenberg & Spagnolo) [20] studies have examined the efficacy of the proposal in reducing the incidents of harassment bribes (also called *extortionary corruption* in the literature). While there was some support [18,19] for the Basu’s proposal of asymmetric liability, reduction in bribery incidents was found to be correlated with the bribe-taker’s inability to retaliate against the bribe-giver. Surprisingly, they also found that refunding the bribe-amount to the bribe giver after prosecution of the bribe-taker in the asymmetric liability scenario does not appear to facilitate reduction in incidents of bribery. The extent to which a citizen’s access to multiple officials (providing the same service) can reduce corruption was investigated by Ryvkin and Serra [19]. They found that such competition between service providing officials facilitated reduction of bribery incidents only if the search cost of finding less corrupt officials were low. A contrasting study on the effect of changing from symmetric to asymmetric punishment in the case of collusive bribes [4] highlighted the major difference between harassment bribes and collusive bribes by showing that asymmetric liability increases incidents of bribery in the latter case. In a different context, evolutionary game theory models have also been used to investigate the effects of corruption of enforcers entrusted to prevent illegal harvesting of forests [21]. However, the scenario discussed falls under the category of collusive bribes where an enforcer can be bribed by harvesters to gain more than their fair share of access to a natural resource.

The controlled experimental studies [18,19] are useful in understanding the impact of symmetric and asymmetric penalties under certain restrictive circumstances. The results of Abbink (2014) are based on the outcome of a single interaction between pairs of individuals who are randomly assigned the roles of officials and citizens. The distribution of strategies across the populations of citizens and officials correspond to a single point in our phase diagrams (see the “Results” section) provided that distribution is considered to be the equilibrium distribution. Hence their results do not shed any light on how the outcomes might change as prosecution rates, penalties imposed and other parameters are varied over a wide range of values. Neither do they address how the evolution of an individual’s behaviour affects the relative abundance of different strategies in the population over time. The theoretical analysis of Dufwenberg and Spagnolo [20] is restricted to finding equilibrium solutions and also does not provide any insight into the dynamical evolution of the system towards equilibrium.

In this paper, we examine Basu’s proposal by constructing deterministic and stochastic evolutionary game-theoretic models of bribery. Evolutionary game-theory was developed to apply game-theoretic models to study biological evolution [22,23]. Since then it has been extensively used to study evolution of cooperation [24], animal behaviour [25] and social conflict in a variety of scenarios. An advantage of evolutionary game-theoretic methods over conventional game theory is that it allows us to examine the dynamical evolution of different strategies in the population and provides a quantitative framework for examining the conditions under which honest strategies can prevail. Moreover, stochastic evolutionary game-theoretic models are useful for understanding the effect of noise arising due to finite population size on the fixation probabilities of different strategies in the population.

Our evolutionary game-theoretic models specify the outcome of interaction between two distinct populations of individuals, one requiring a service (citizens) and the other providing the service (officers), through a payoff matrix. We first describe a deterministic five strategy model that allows us to compare the outcome of the symmetric liability scenario with the asymmetric liability scenario proposed by Basu. Analysis of the results of this model suggests that when prosecution rates are low, citizens who refuse to pay a bribe do not have any significant impact on the equilibrium distribution of strategies in the population. To better understand the conditions under which the asymmetric liability scenario is effective in reducing incidents of bribery, we analyse two four-strategy models that are distinguished by differences in rules according to which an individual’s strategy is updated over time.

The effect of varying the bribe amount, penalty for taking bribes, prosecution rate and cost of complaining on the equilibrium population of different strategies is investigated for both symmetric and asymmetric liabilities. We find that the asymmetric liability scenario facilitates significant reduction in bribery incidents compared to the symmetric liability scenario under generic circumstances. However, when bribe amount and a citizen’s cost of complaining about the bribery incident are high, prosecution rates and penalty for taking bribes are low; even the asymmetric liability scenario fails to stem the tide of corruption. Moreover, the mechanisms according to which individuals change their strategies over time (strategy update rules) also impair the effectiveness of the asymmetric liability scenario in reducing bribery.

## Results

**Fig 1:**
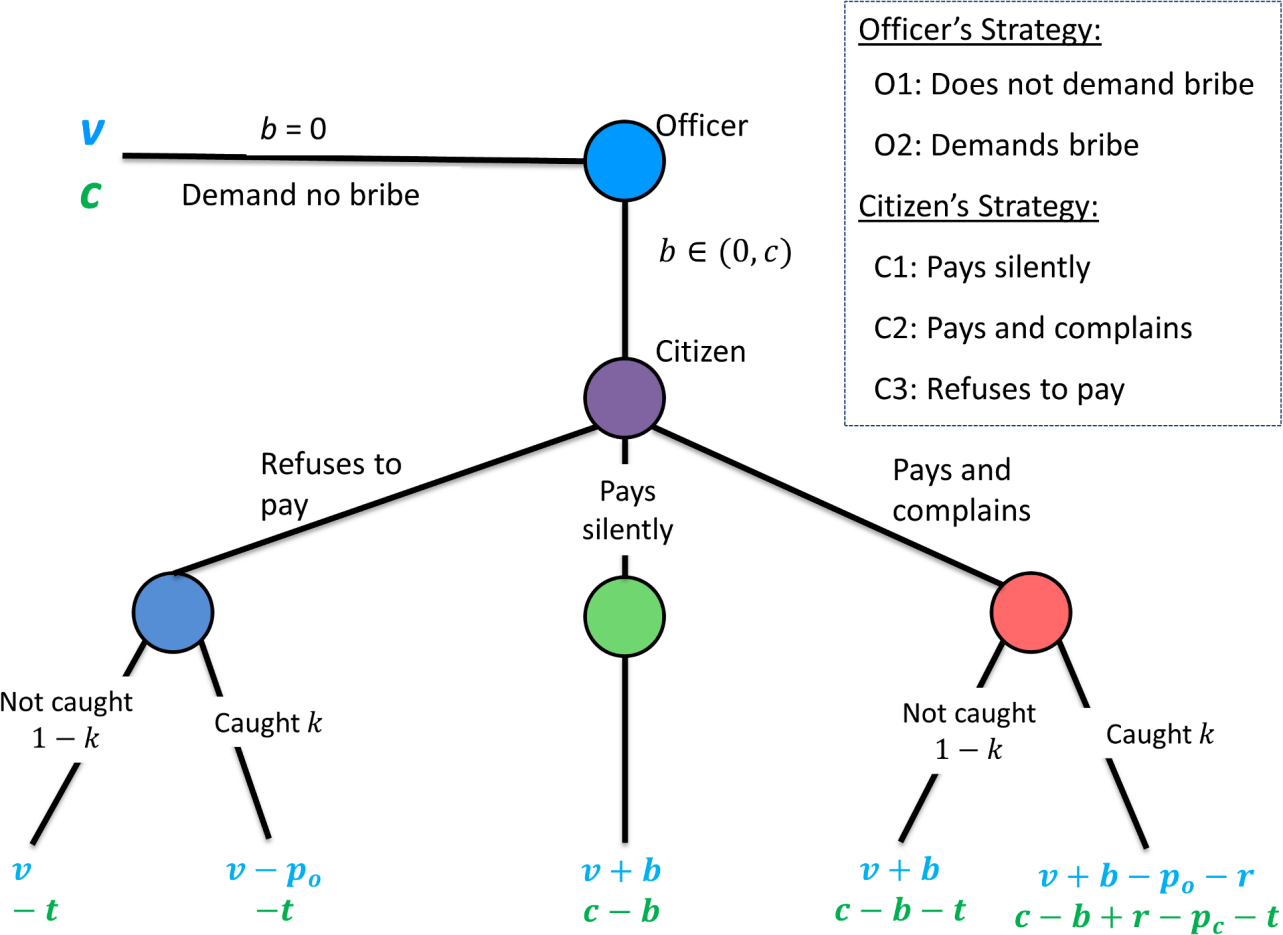
Game Tree for the harassment bribery game. Condition for (a) Symmetric liabilities: *p*_*o*_ = *p*_*c*_ (b) Asymmetric liabilities: *p*_*o*_ > 0 and *p*_*c*_ = 0.

A bribery scenario can be modeled as an asymmetric sequential game between citizens and officers. Each group of citizens and officers is further sub-divided on the basis of the strategy employed by the members. Hence, there are officers who do not demand bribes (*O*_*1*_) and those who do demand a bribe (*O*_*2*_). Similarly, citizens are sub-divided on the basis of their response to a bribe demand. A citizen can pay silently without complaining about the bribery incident (*C*_*1*_) and pay but then register a complaint in the appropriate forum (*C*_*2*_). If the officer does not demand a bribe, the citizen gets a payoff of *c* which is the cost of the service, irrespective of the strategy she follows and the officer receives a fixed payoff of *v* which can be thought of as the salary of the officer. If the officer demands a bribe (*O*_*2*_), a citizen belonging to the sub-category *C*_*1*_ gets a payoff of *c−b* and the officer gets a payoff of *v+b*. If a citizen decides to pay the bribe and complain (*C*_*2*_), she has to bear a cost of complaining *t* which can be attributed to the cost involved in litigating the social conflict. A citizen can “refuse to pay a bribe” (*C*_3_) when interacting with a corrupt official. If the officer demands a bribe *b* (< *c*) and the concerned citizen refuses to pay bribe, the officer gets a fixed payoff of *v* and citizen (*C*_3_) gets nothing. Not all complaints lead to prosecution of the accused officer. The probability of prosecution depends upon the efficiency of justice delivery system. The probability of the prosecution is given by the parameter *k* (0 < *k* < 1). When prosecuted, the corrupt officer is punished with penalty *p*_*o*_ and citizen with *p*_*c*_. When a bribery incident is discovered which happens with probability *k*, citizens are refunded an amount *r*. In the symmetric liability scenario, since both the citizen and the officer are equally liable for the crime and hence prosecuted with equal punishment (*p*_*o*_ = *p*_*c*_), bribe-giving citizens do not have any incentive to complain. In the asymmetric liability scenario, however, officers are more liable and therefore penalized more than the citizen (*p*_*o*_ > *p*_*c*_). The possible interactions are represented by the game tree depicted in Fig 1.

### 2.1 Replicator Dynamics for Five Strategy Model

In this model there are three strategies available to the citizen who interacts with a corrupt officer. The outcome of the other interactions remains the same as described in the previous sub-section. The payoff matrix of game is:

$$\begin{array}{l} \begin{array}{ccc} & & \begin{array}{cccc} & \;O_{1} & & \begin{array}{ccc} & \;O_{2} & \end{array} \end{array} \end{array}\\ M_{1}=\begin{array}{c} C_{1}\\ \begin{array}{c} C_{2}\\ C_{3} \end{array} \end{array}\left(\begin{array}{cc} c & c-b\\ \begin{array}{c} c\\ c \end{array} & \begin{array}{c} c-b-t+k(r-p_{c})\\ -t \end{array} \end{array}\right) \end{array}$$ (1)

$$\begin{array}{l} \begin{array}{cccc} & & & \begin{array}{cccc} \begin{array}{cccc} \begin{array}{cccc} \;C_{1} & & & \;C_{2} \end{array} & & & \end{array} & \;C_{3} & & \end{array} \end{array}\\ M_{2}=\begin{array}{c} O_{1}\\ O_{2} \end{array}\left(\begin{array}{cc} v & \begin{array}{ccc} \begin{array}{ccc} \begin{array}{ccc} & v & \end{array} & & \end{array} & \begin{array}{cc} & \;v \end{array} & \end{array}\\ v+b & \begin{array}{cc} v+b-k(p_{o}+r) & v-kp_{o} \end{array} \end{array}\right) \end{array}$$ (2)

We start with a population of citizens and officers and study the evolving population dynamics by analysing a coupled set of replicator equations. The interaction between officer and citizens occurs at random and after each interaction both the players gets some payoff. A randomly chosen player in each of the two groups then compares their strategy with another randomly chosen individual belonging to the same group and imitates the strategy of the latter individual with a probability proportional to the difference between the payoffs of two players. It can be shown [26,27] that in the infinite population (deterministic) limit, such a local update rule leads to the familiar replicator equation.

In a well-mixed population of officers and citizens only the interaction of citizens with officers and officer with citizens yields non-zero payoffs as given by the payoff matrix (1) and (2). The expected payoff of different strategies of citizens and officers is then given by *π*_*Ci*_ = *x*_*O*1_ *M*_1_(*i*,1) + *x*_*O*2_ *M*_1_(*i*,2) and *π*_*Oi*_ = *x*_*C*1_ *M*_2_(*i*,1) + *x*_*C*2_ *M*_2_(*i*,2) + *x*_*C*3_ *M*_2_(*i*,3) respectively. Here *x*_*C*1_,*x*_*C*2_,*x*_*C*3_,*x*_*O*1_ and *x*_*O*2_ are the frequency of strategies *C*_*1*_, *C*_*2*_, *C*_*3*_, *O*_*1*_ and *O*_*2*_ respectively. The general replicator equations giving the time evolution of frequencies of officers and citizens are ${\dot{x}}_{Oi}=(\pi_{Oi}-\phi_{O})x_{Oi}$ and ${\dot{x}}_{Ci}=(\pi_{Ci}-\phi_{C})x_{Ci}$ respectively. Here *ϕ*_*O*_ and *ϕ*_*C*_ are the average expected payoff of citizens and officers as defined by *ϕ*_*O*_ = *π*_*O*1_ *x*_*O*1_ + *π*_*O*2_ *x*_*O*2_ and *ϕ*_*C*_ = *π*_*C*1_ *x*_*C*1_ + *π*_*C*2_ *x*_*C*2_ + *π*_*C*3_ *x*_*C*3_. The coupled set of replicator equations then simplifies to

$${\dot{x}}_{O1}=x_{O1}x_{O2}[kp_{o}(1-x_{C1})+krx_{C2}-b(x_{C1}+x_{C2})]$$ (3)

$${\dot{x}}_{O2}=x_{O1}x_{O2}[b(x_{C1}+x_{C2})-kp_{o}(1-x_{C1})-krx_{C2}]$$ (4)

$${\dot{x}}_{C1}=x_{C1}x_{O2}[(c-b)x_{C3}+t(1-x_{C1})+k(p_{c}-r)x_{C2}]$$ (5)

$${\dot{x}}_{C2}=x_{C2}x_{O2}[(c-b)x_{C3}-tx_{C1}-k(p_{c}-r)(1-x_{C2})]$$ (6)

$${\dot{x}}_{C3}=x_{C3}x_{O2}[k(p_{c}-r)x_{C2}-tx_{C1}-(c-b)(1-x_{C3})]$$ (7)

The fixed population of the citizens and officer gives us two additional constraints on the frequency of strategies: *x*_*C*1_ + *x*_*C*2_ + *x*_*C*3_ = 1 & *x*_*O*1_ + *x*_*O*2_ = 1.

**Fig 2:**
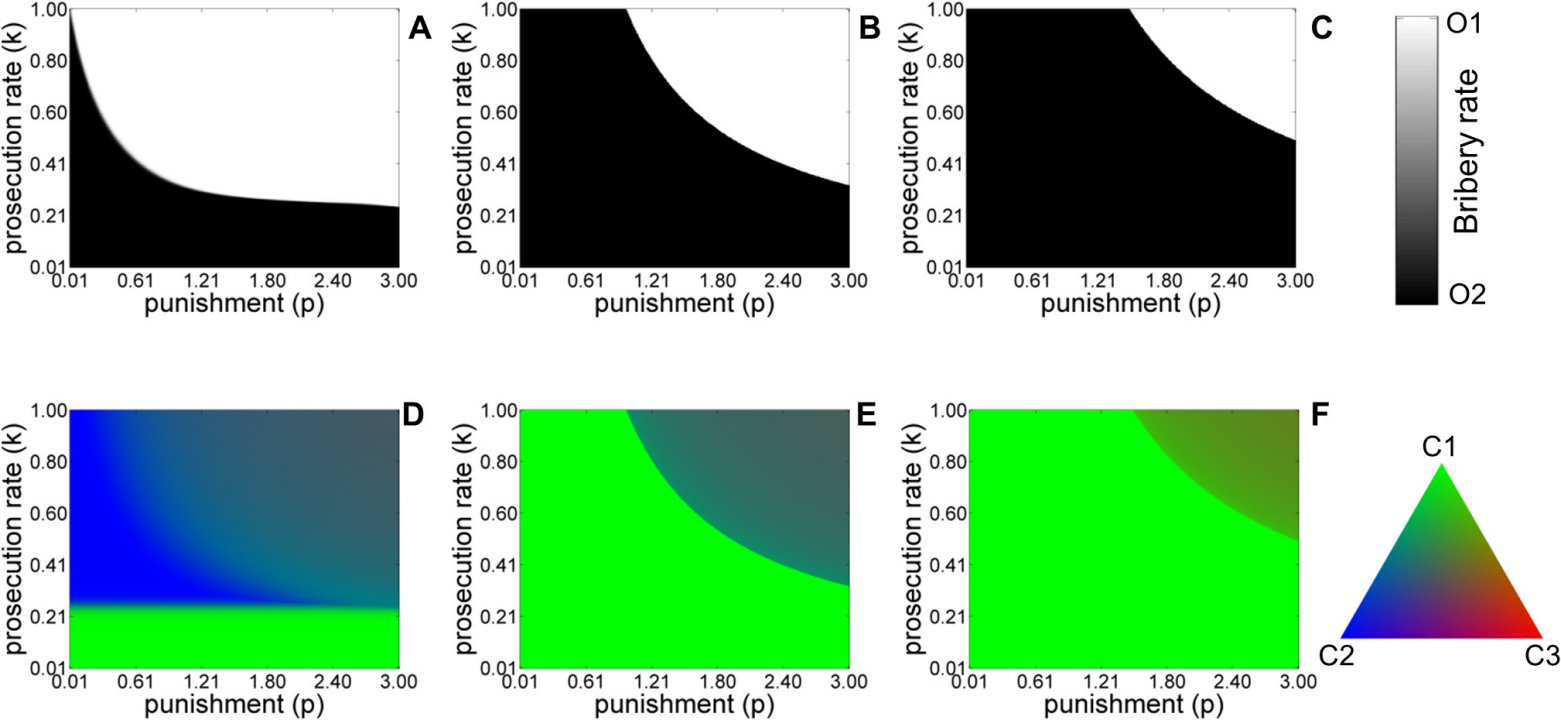
Equilibrium population structure for the five-strategy model for variable punishment *p*, and prosecution rate *k* ‘with refund’ (A, D) and ‘without-refund’ (B, E) in the asymmetric liability and symmetric liability scenarios (C, F). Shades of white and black colors denote the equilibrium abundance of officers of type *O*_1_ and *O*_2_. Shade of green and blue and red colors denote the stationary frequencies of *C*_1_, *C*_2_ and *C*_3_ categories of citizens. The values of other parameters are *c* = 1, *v* = 1, *b* = 0.4, *t* = 0.1. The initial condition corresponds to *x*_*C*1_ = 1/3, *x*_*C*2_ = 1/3, *x*_*C*3_ = 1/3, *x*_*O*1_ = 1/2, *x*_*O*2_ = 1/2 i.e. all strategies are initially equally abundant in the population.

**Fig 3:**
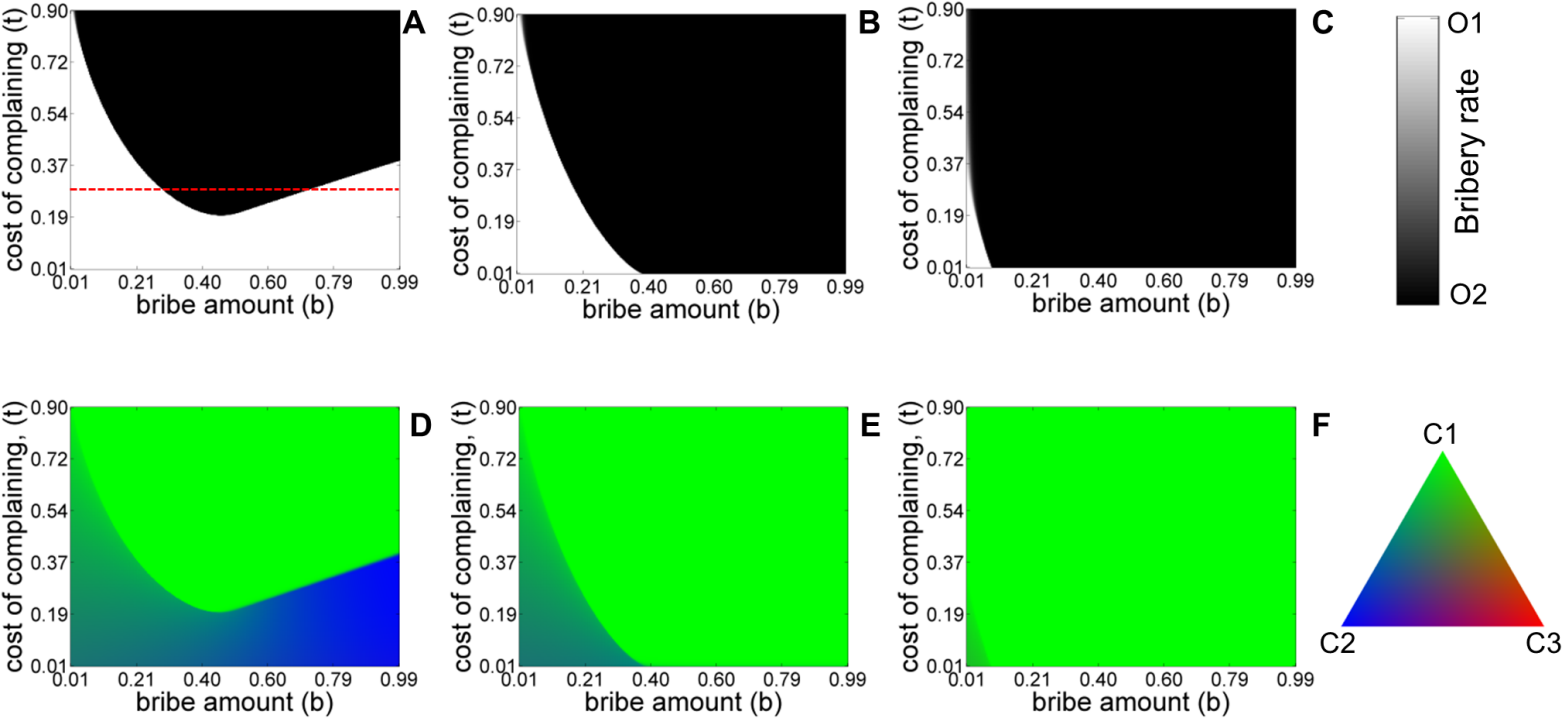
Equilibrium population structure for the five-strategy model as a function of bribe amount *b*, and cost of complaining *t*, ‘with refund’ (A, D) and ‘without-refund’ (B, E) in the asymmetric liability and symmetric liability scenarios (C, F). Shades of white and black colors denote the equilibrium abundance of officers of type *O*_1_ and *O*_2_. Shade of green and blue and red colors denote the stationary frequencies of *C*_1_, *C*_2_ and *C*_3_ categories of citizens. The values of other parameters are: *c* = 1, *v* = 1, *p*_*o*_ = 2 *k* = 0.4. The initial condition corresponds to *x*_*C*1_ = 1/3, *x*_*C*2_ = 1/3, *x*_*C*3_ = 1/3, *x*_*O*1_ = 1/2, *x*_*O*2_ = 1/2.

**Fig 4:**
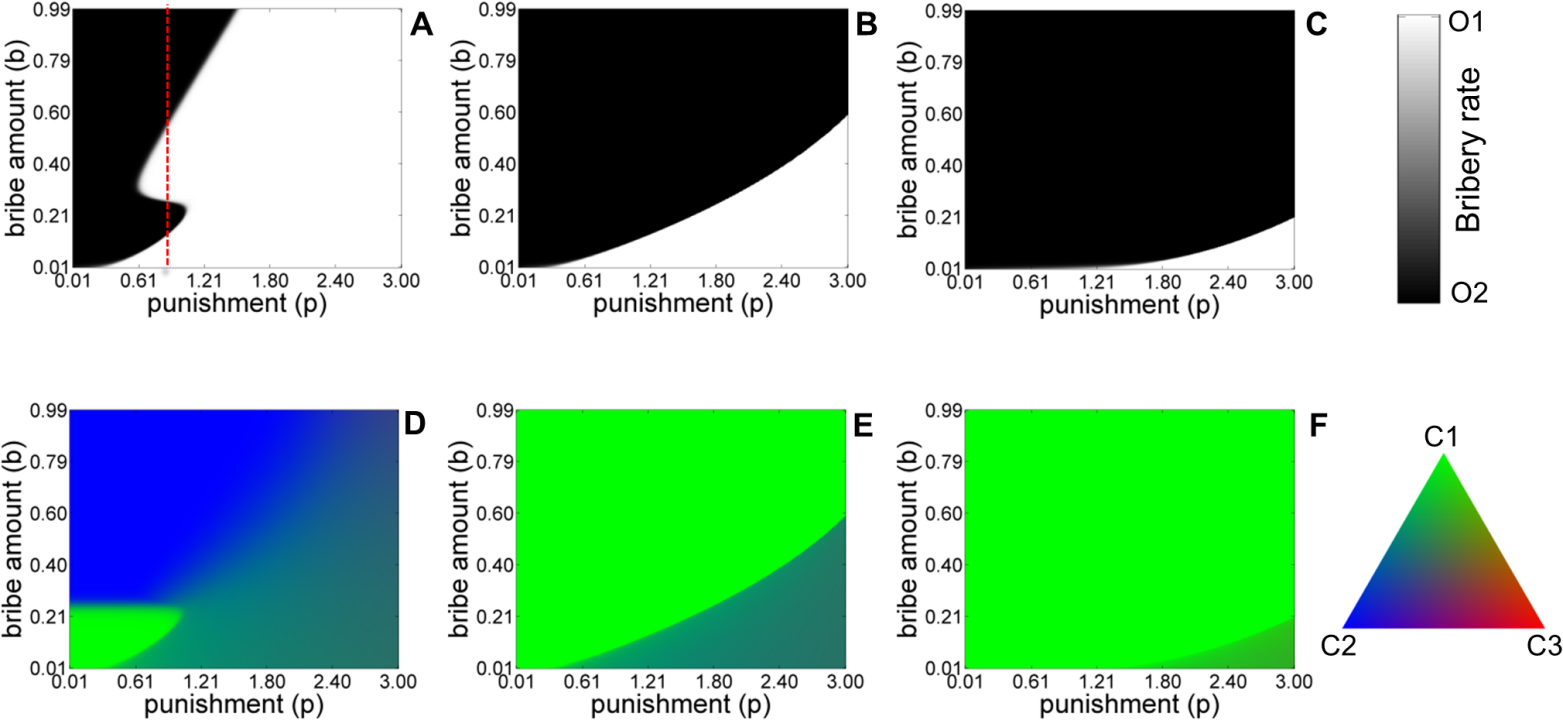
Equilibrium population structure for the five-strategy model as a function of punishment *p*, and bribe amount *b* in ‘with refund’ (A, D) and ‘without-refund’ (B, E) asymmetric liability and symmetric liability scenario (C, F). Shades of white and black colors denote the equilibrium abundance of *O*_1_ and *O*_2_ type of officers. Shade of green and blue and red colors denote the stationary frequencies of *C*_1_, *C*_2_ and *C*_3_ categories of citizens. The values of other parameters are: *c* = 1, *v* = 1, *k* = 0.4, *t* = 0.1. The initial condition corresponds to *x*_*C*1_ = 1/3, *x*_*C*2_ = 1/3, *x*_*C*3_ = 1/3, *x*_*O*1_ = 1/2, *x*_*O*2_ = 1/2.

Figs 2–4 show the phase diagrams for the equilibrium population structure for different scenarios. Fig 2 shows the effect of varying the punishment for taking bribes and the prosecution rate for asymmetric liability with (panel A, D) and without (panel B, E) refund and symmetric liability (panel C, F). In the asymmetric liability scenario, we find evidence of a sharp transition from a corrupt to a bribe-free society as either penalty for taking bribes or prosecution rate is increased. Allowing the bribe amount to be refunded increases the incentive for citizens to complain. This has the effect of shifting the phase boundary resulting in the transition to the bribe-free society occurring for lower values of punishment and prosecution rate (compare panels A, D (with refund) with B, E (no refund) in Fig 2). Since there cannot be any refund in the symmetric liability scenario, the transition to a bribe-free society is possible only for even higher penalties and prosecution rates (panel C, F in Fig 2). The phase diagrams for the officers (panels A-C) and citizens (panels D-F) are clearly correlated. The predominance of citizens who are willing to pay a bribe affects the fraction of corrupt officers in the population and vice versa. In the symmetric liability scenario, the presence of citizens, who refuse to pay a bribe even in small numbers can aid in the transition to a bribe-free society only for large values of penalty, prosecution rates and small values of bribe amounts, cost of complaining (figure not shown).

For the case of asymmetric liability ‘*with refund*’ (panel A and D), we find that for certain fixed values of the cost of complaining parameter (for example, see the red dashed line in ‘Fig 3A’), *O*_2_ needs to optimize the value of bribe demand *b* to survive in the population. If the bribe demand is either too low or too high, *O*_2_ cannot be sustained in the population. Too small a bribe amount does not yield a sufficient payoff advantage to the corrupt officers over their honest counterparts as a result of which the former cannot be sustained in the population. However, rational choice models suggest that corrupt officers should demand the maximum possible bribe that is consistent with the cost of the service. This conclusion is valid in the *asymmetric liability* without refunds and in the *symmetric liability* (panels B, E and C, F) scenarios but in the case of *asymmetric liability with refund* we find the interesting result that too large a bribe-demand also leads to the elimination of the corrupt officers. The result can also be understood from the Eqs (1)–(5) given above. When the bribe amount demanded is large, it increases the payoff advantage that corrupt officers have over honest officers. However, since the *r = b* in our model, the hope of getting a refund increases the incentive for complaining citizens which results in an increase in their number. The increase in the latter offsets the advantage of corrupt officers and eventually allows honest officers to prevail. This is also evident from the increasing intensity of blue seen in panel D as the bribe amount is increased for a fixed but moderate value of *t*. For intermediate values of *b*, the increase in the number of complaining citizens is not sufficient enough to overcome the advantage that corrupt officers have from demanding a bribe. Hence, this allows the corrupt officers to eventually get fixed in the population. Existence of *C*_3_ in the population does not have any qualitative effect on the equilibrium population in asymmetric liability scenarios with and without refund (Fig 3 panels A, D and B, E). In symmetric liability scenario, the presence of *C*_3_ is the major factor in ensuring that honest officers (*O*_1_) get fixed in the population for very low value of bribe demand and low cost of complaining.

For the symmetric liability case (Fig 4C and 4F) honest officers can get fixed in the population only when the punishment is high and bribe amount demanded is low. When the bribe amount demanded was high, the penalty for taking bribes had to be increased accordingly to eliminate corrupt officers from the population. Similar observations can also be made for the asymmetric liability scenario *without* refunds (Fig 4B and 4E). However, in the latter case, the elimination of corrupt officials occurs for significantly lower values of punishment for low to moderate bribe demands. This is due to the fact that in asymmetric liability case the presence of citizens who pay and complain (*C*_2_) along with those who refuse to pay bribes (*C*_3_) work together to reduce the payoff advantage of the corrupt officers thereby leading to their eventual elimination. When refund is allowed (Fig 4A and 4D) an interesting feature of the dynamics is manifest through the shape of the phase boundary at equilibrium. For a small range of values of punishment (*p*), the system goes through multiple transitions as *b* is steadily increased for fixed *p*_*0*_ (see red dashed line). There is a small range of values of *b* for which the corrupt officers thrive in the population. An initial increase in b increases the payoff advantage that corrupt officers have relative to honest officers leading to the fixation of the former in the population. In this regime, the refund offered turns out to be insufficient to increase the number of complaining citizens and the population is therefore dominated by citizens who pay silently (see panel D). Further increase in b increases the frequency of complaining citizens since *b* is correlated with *r* in our model. This in turn reduces the advantage of corrupt officers and lead to their elimination from the equilibrium population. As *b* continues to increase, the number of complaining citizens eventually saturates and the payoff advantage arising from a large bribe demand is sufficient to again lead to the fixation of corrupt officers in the population. This accounts for the third transition when b is further increased for fixed *p*_*0*_. However, as *p*_*0*_ increases, the payoff advantage even for large bribe amounts is not enough to offset the high cost of being caught (which also occurs with a higher probability) and this leads to eventual elimination of corrupt officers.

### 2.2 Replicator Dynamics for Four Strategy Model

If the probability of prosecution of the bribery incident reported by *C*_*3*_ is very low, then the citizens of type *C*_*3*_ fails to play any significant role in the population dynamics of the system. (The corresponding game tree showing the various interactions in the four strategy model is given in S1 Fig) The evolution of the population is then described by the set of four coupled replicator equations for the time evolution of frequencies of the officers and citizens. We start with two fixed populations of citizens and officers. Officers interact only with citizens and vive-versa. The evolutionary dynamics would then determine the extent to which citizens who pay silently can compete with citizens who pay and complain. The outcome would in turn determine whether it is more favourable for honest officers to increase in frequency. The payoff matrix for the citizens and officers in this model can be written as:

The payoff matrix for the citizens and officers in this model can be written as:

$$\begin{array}{l} \begin{array}{ccc} & & \begin{array}{cccc} & \;O_{1} & & \begin{array}{ccc} & \;O_{2} & \end{array} \end{array} \end{array}\\ M_{3}=\begin{array}{c} C_{1}\\ C_{2} \end{array}\left(\begin{array}{cc} c & c-b\\ c & c-b-t+k(r-p_{c}) \end{array}\right) \end{array}$$ (8)

$$\begin{array}{l} \begin{array}{ccc} & & \begin{array}{cccc} & \;C_{1} & & \begin{array}{ccc} & \;C_{2} & \end{array} \end{array} \end{array}\\ M_{4}=\begin{array}{c} O_{1}\\ O_{2} \end{array}\left(\begin{array}{cc} v & v\\ v+b & v+b-k(p_{o}+r) \end{array}\right) \end{array}$$ (9)

The replicator equations for the four strategy model simplifies to:

$${\dot{x}}_{C1}=(t-kr+kp_{c})x_{C1}x_{C2}x_{O2}$$ (10)

$${\dot{x}}_{C2}=(kr-kp_{c}-t)x_{C1}x_{C2}x_{O2}$$ (11)

$${\dot{x}}_{O1}=(k(p_{o}+r)x_{C2}-b)x_{O1}x_{O2}$$ (12)

$${\dot{x}}_{O2}=(b-k(p_{o}+r)x_{C2})x_{O1}x_{O2}$$ (13)

Here it is assumed that the population of both officers and citizens is fixed and has been normalized to unity *x*_*C*1_ + *x*_*C*2_ = 1 and *x*_*O*1_ + *x*_*O*2_ = 1.

A useful way to analyze the system of Eqs (10)–(13) is to vary two parameters of interest and ascertain how the equilibrium distribution of various strategies changes. In particular we are interested in exploring the conditions under which honest officers are predominant and when the stationary distribution is characterized by low frequency of honest officers in the population.

**Fig 5:**
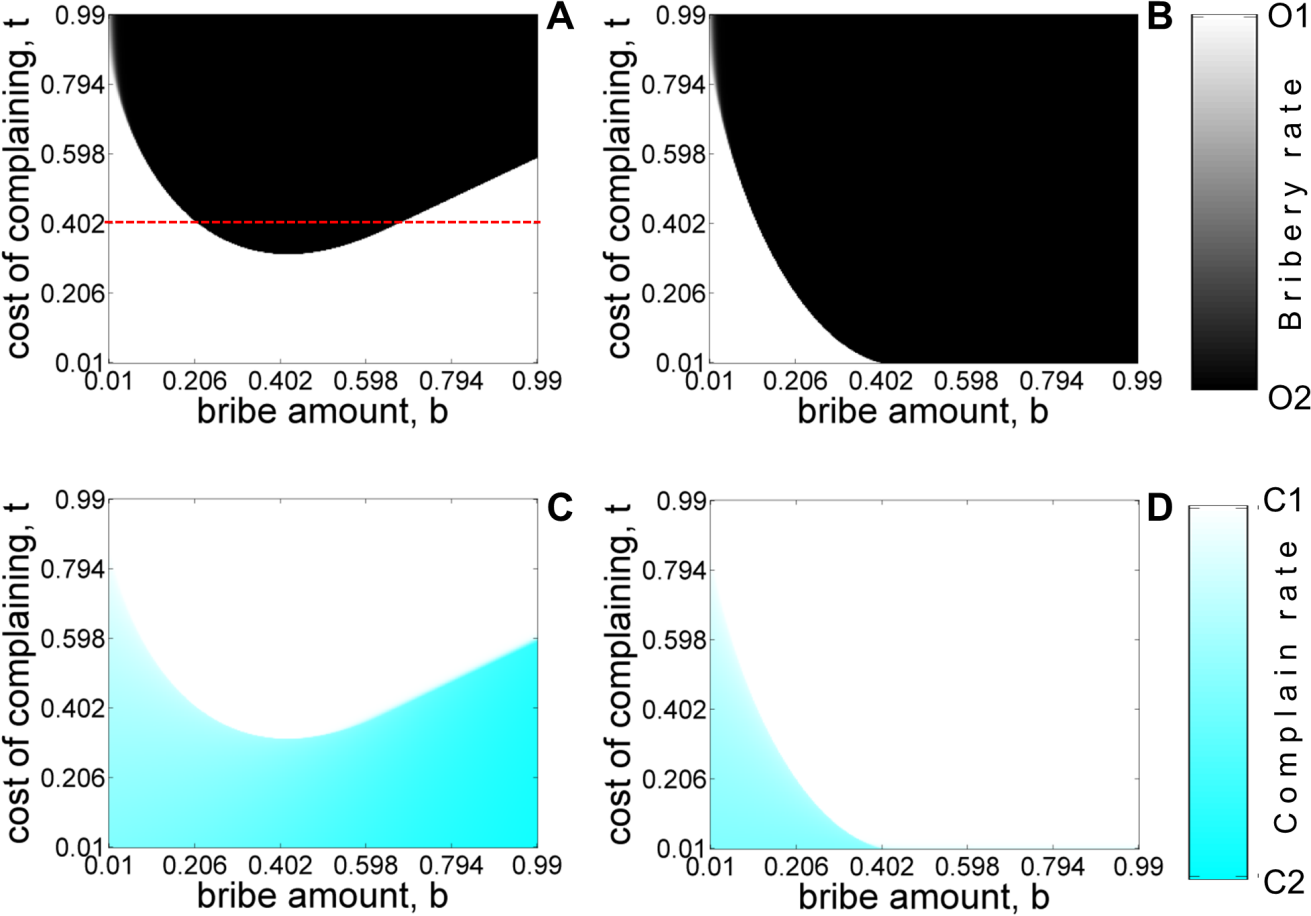
Equilibrium population for the four-strategy model while varying of bribe amount *b*, and cost of complaining *t* with refund (A, C) and without-refund (B, D) for asymmetric liability scenario. Shades of white and black colors denote the equilibrium abundance of *O*_1_ and *O*_2_ type of officers. Shade of white and cyan colors denote the stationary frequencies of *C*_1_ and *C*_2_ type of citizens. The values of other parameters are: *c* = 1, *v* = 1, *p*_*o*_ = 2, *k* = 0.6. The initial condition corresponds to *x*_*C*1_ = 0.5, *x*_*C*2_ = 0.5, *x*_*O*1_ = 0.5, *x*_*O*2_ = 0.5

**Fig 6:**
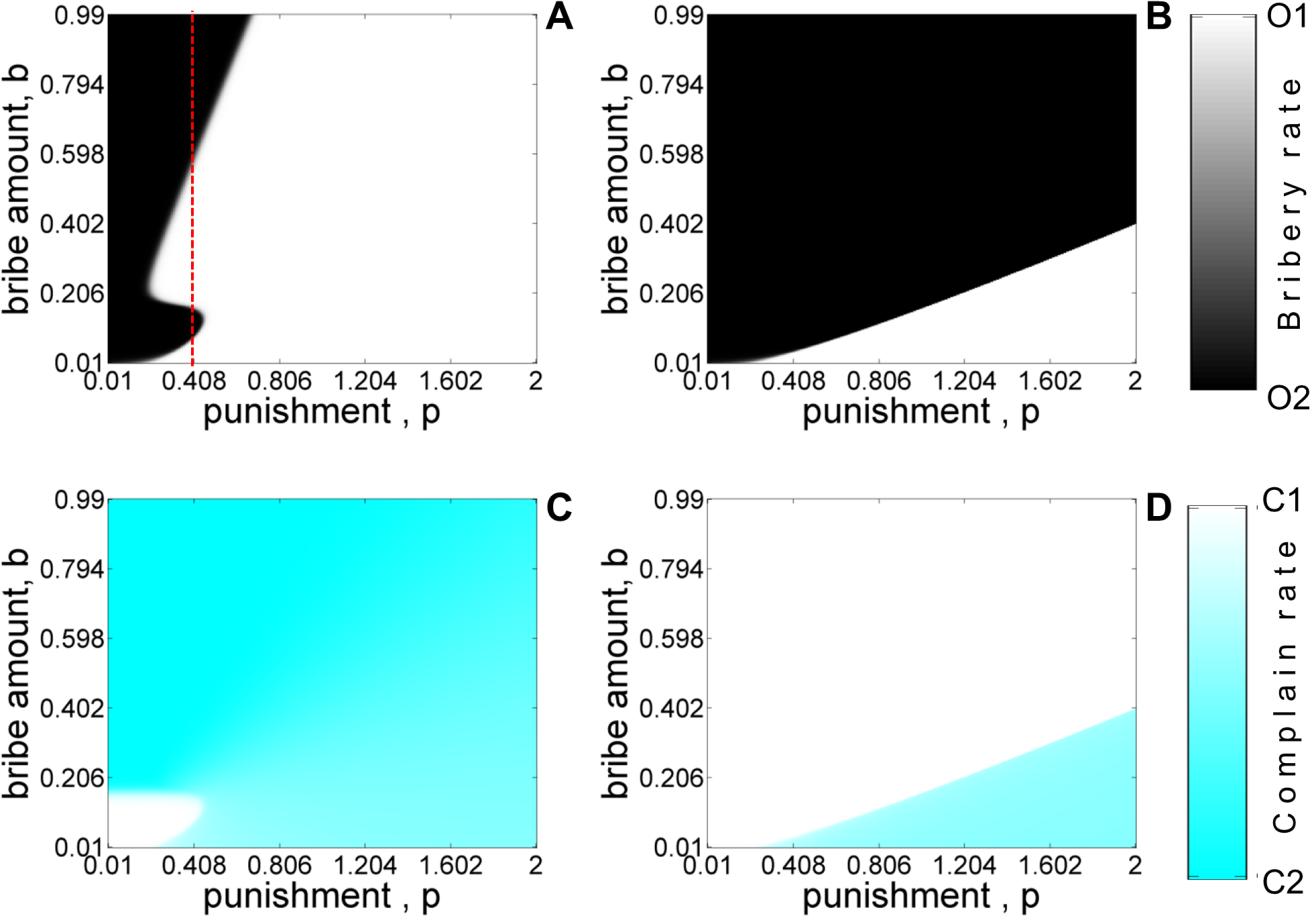
Equilibrium population for the four-strategy model as a function of bribe amount *b*, and punishment *p* with refund (A, C) and without-refund (B, D) for asymmetric liability scenario. Shades of white and black color denote the equilibrium abundance of *O*_1_ and *O*_2_ type of officers. Shades of white and cyan color denote the stationary frequencies of *C*_1_ and *C*_2_ type of citizens. The values of other parameters are: *c* = 1, *v* = 1, *k* = 0.6. The initial condition corresponds to *x*_*C*1_ = 0.5, *x*_*C*2_ = 0.5, *x*_*O*1_ = 0.5, *x*_*O*2_ = 0.5

**Fig 7:**
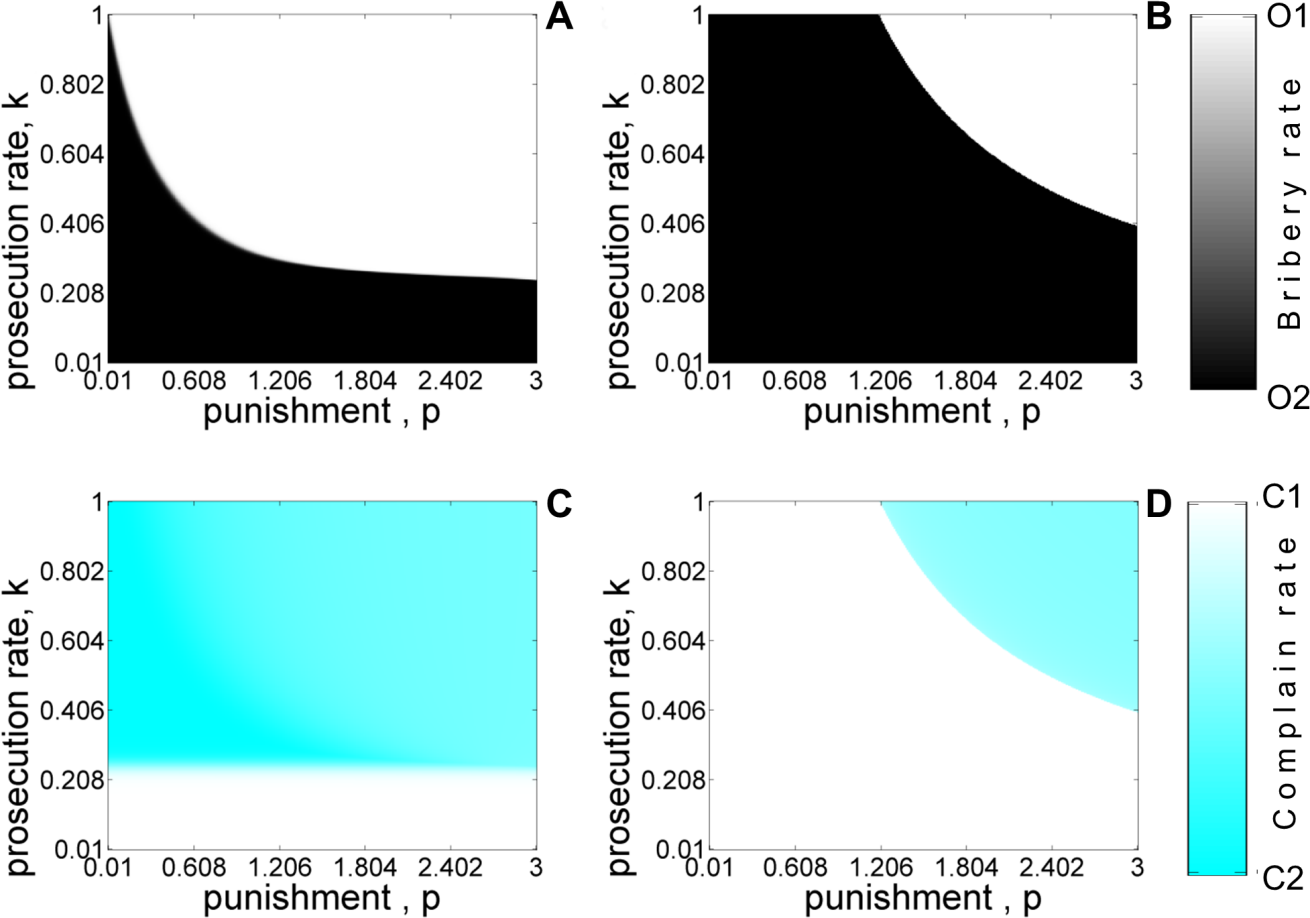
Equilibrium population for the four-strategy model as a function of punishment *k* and prosecution rate *k* with refund (A, C) and without-refund (B, D) for asymmetric liability scenario. Shades of white and black color denote the equilibrium abundance of *O*_1_ and *O*_2_ type of officers. Shades of white and cyan color denote the stationary frequencies of *C*_1_ and *C*_2_ type of citizens. The values of other parameters are: *c* = 1, *v* = 1, *b* = 0.4, *t* = 0.1. The initial condition corresponds to *x*_*C*1_ = 0.5, *x*_*C*2_ = 0.5, *x*_*O*1_ = 0.5, *x*_*O*2_ = 0.5.

**Fig 8:**
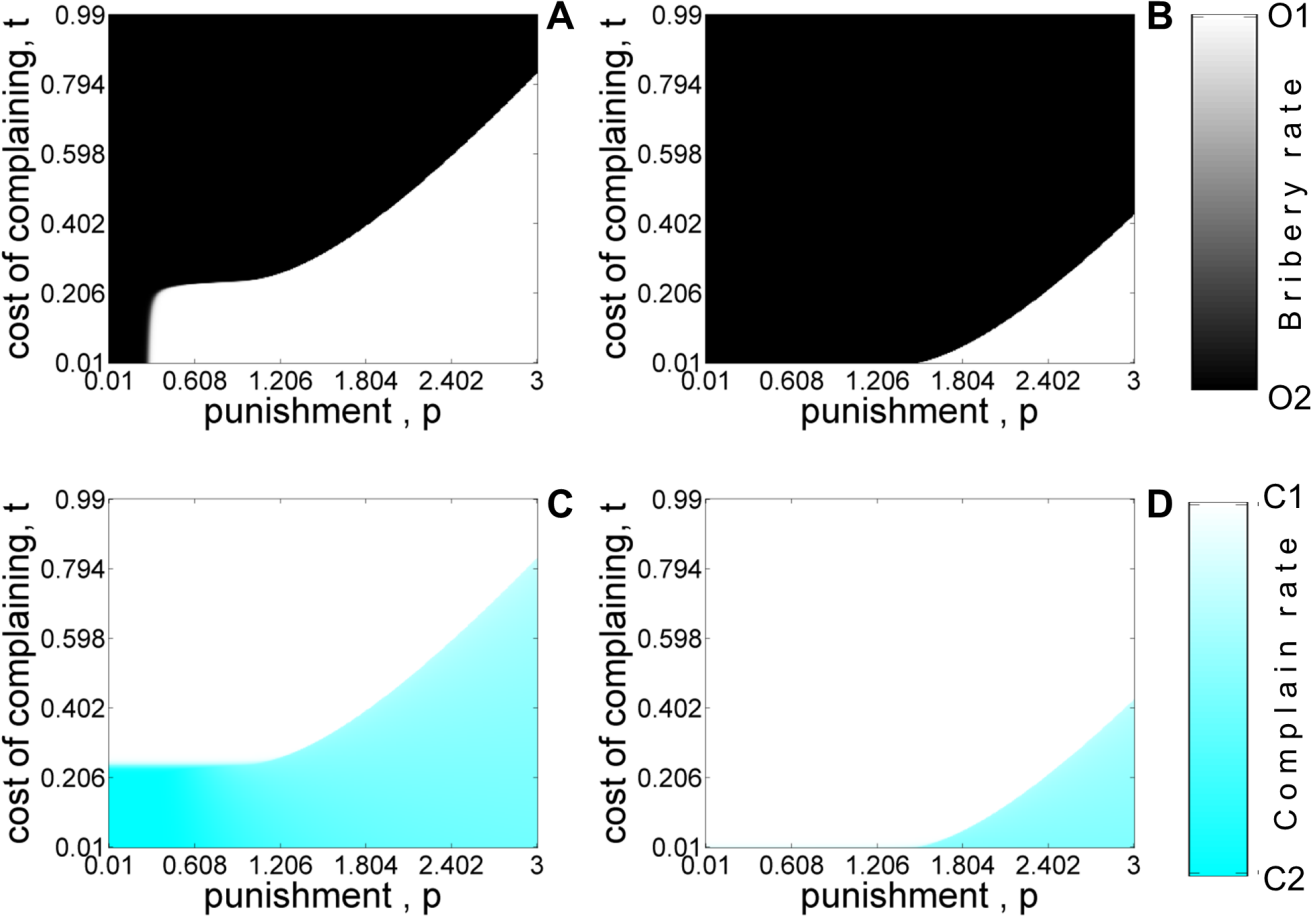
Equilibrium population for the four strategy model as a function of punishment *p* and cost of complaining *t* with refund (A, C) and without-refund (B, D) for asymmetric liability scenario. Shades of white and black color denote the equilibrium abundance of *O*_1_ and *O*_2_ type of officers. Shades of white and cyan color denote the stationary frequencies of *C*_1_ and *C*_2_ type of citizens. The values of other parameters are: *c* = 1, *v* = 1, *k* = 0.6, *b* = 0.4. The initial condition corresponds to *x*_*C*1_ = 0.5, *x*_*C*2_ = 0.5, *x*_*O*1_ = 0.5, *x*_*O*2_ = 0.5.

Figs 5–8 shows the equilibrium population structure with (panels A,C) and without (panels B,D) refund as various parameters of interest are varied. The results are similar to the ones obtained using the five-strategy model. When ‘*refunds*’ are allowed, *O*_2_ needs to calibrate the bribe demand *b* for certain values of ‘cost of complaining’ (*t*) in order to be sustained in the population. Fig 6 shows the phase diagram of different strategies at equilibrium as the bribe amount and punishment are varied. The plot shows the same qualitative features observed in Fig 4. Fig 7 and Fig 8 shows the equilibrium phase diagram as the prosecution rate (*k*) and cost of complaining (*t*) are varied with punishment (*p*_*o*_). From these figures, we again clearly see that refunds (panels A,C) can act as an incentive for citizens to report corrupt officers and the consequent increase in the number of complaining citizens can lead to fixation of honest officers for smaller values of *p*_*0*_ even for moderate values of *k* and *t*.

**Fig 9:**
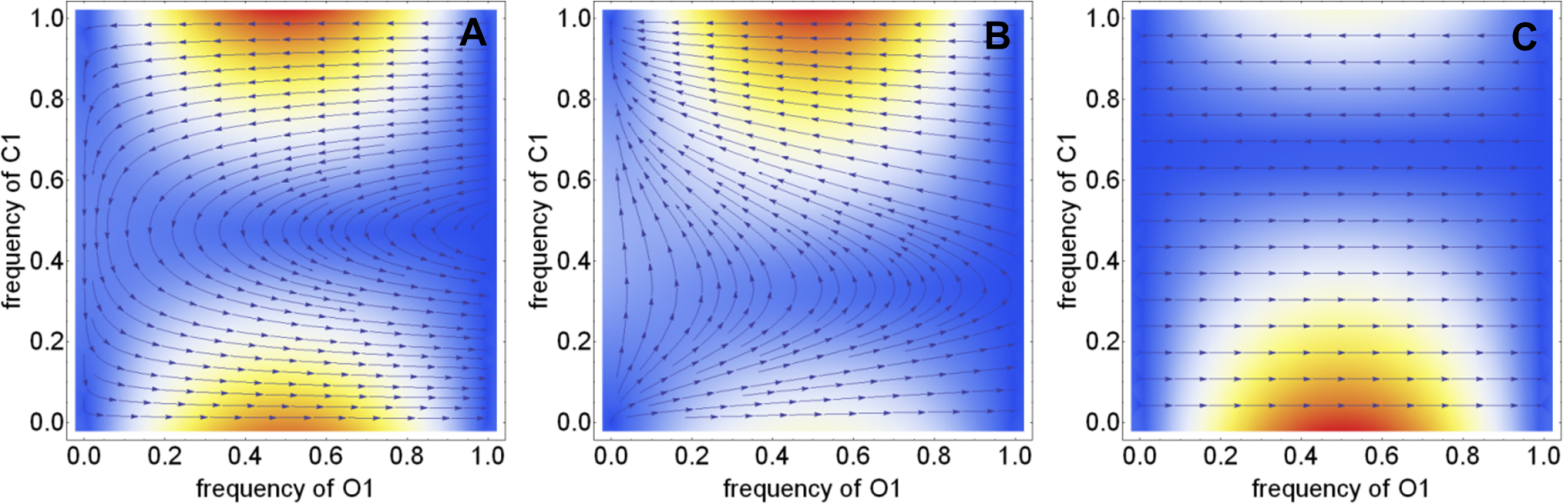
Phase diagram of the four-strategy model with asymmetric liability for variable initial conditions. Panel A-C corresponds to situations ‘with refund’, ‘without refund’ and ‘no complaining cost without refund’ respectively. Each point in this phase plot (simplex) specifies the population structure of officers and citizens. Arrows represents the direction of the change in frequency of a strategy in the phase space. Red represents fast dynamics and blue represents slow dynamics, close to fixed points. The values of the parameters are: *c* = 1, *v* = 1, *p*_*o*_ = 1.5, *k* = 0.4, *b* = 0.4, *t* = 0.1 for panel A and B. Parameter values are: *c* = 1, *v* = 1, *p*_*o*_ = 3, *k* = 0.4, *b* = 0.4, *t* = 0 for panel C.

It is also useful to explore the effect of changing the initial conditions (initial fraction of different strategies in the population) on the equilibrium population structure in the asymmetric punishment model proposed by Basu. Such an analysis reveals the extent to which the initial fraction of honest officers and complaining citizens aids in the eventual fixation of honest strategies in the population. In this context, we were particularly interested in comparing the scenario in which the bribe amount is refunded in full to the citizen following a prosecution to the scenario in which the complaining citizen does not receive any refund. In the former case, we find that unless the frequency of apathetic citizens who pay silently is nearly unity, the equilibrium population structure always converges to a state characterized by the presence of honest officers only (see Fig 9A). However the situation changes when the amount refunded is less than the bribe amount. In that scenario, the eventual fixation of honest officers strongly depends on the initial frequency of apathetic citizens in the population. If the initial fraction of apathetic citizens is higher than a threshold, honest officers are eventually eliminated from the population regardless of their initial fraction in the population. A similar trend is observed in the absence of refunds (*r* = 0) but with a lower threshold (see Fig 9B). These results reinforce the belief that refunds can act as an incentive for citizens to lodge a complaint against corrupt officers.

Another major bottleneck in the fight against corruption lies in the difficulty citizens’ encounter in reporting and seeking redressal against corrupt officials. This is reflected in our model in the high value of the cost of complaining parameter. It has been noted (http://www.ipaidabribe.com/) that sometimes just highlighting incidents of bribery in the public domain and bringing those incidents to the attention of officials in the concerned department can be an effective tool in combating corruption. In the absence of legal actions, such reporting cannot by its very nature compensate the bribe-giver in the asymmetric liability model. Nevertheless, by drastically reducing the cost of complaining and relying on the perceived threat of public outing of the corrupt official, such a scenario can change the outcome of the bribery dynamics. We investigated such scenarios in our model by setting both refund and cost of complaining to zero in the asymmetric liability model. If the initial frequency of apathetic citizens who pay silently is less than 70% regardless of the initial fraction of honest officers, it is possible for honest officers to spread through the population (see Fig 9C). Above the threshold, honest officers are eliminated from the population and the system converges to the equilibrium fixed point associated with the presence of only corrupt officers in the population. The threshold depends on the prosecution rate (*k*) with the fixation of honest officers taking place only when the prosecution rate is maintained above a critical value. This suggests that reducing the cost of complaining alone cannot lead to reduction in corruption if bribe-taking is not adequately disincentivized through by prosecution of corrupt officials.

### 2.3 Alternative strategy exploration model

In the previous models, the sub-populations of officers and citizens had independent roles. However, in reality, officer’s also need access to the set of services that are available to citizens and occasionally will need to interact with other officers controlling the access to such services. To take this factor into account, we distinguish officers (*O*_*ij*_) by two traits denoted by indices *i* and *j*, the first one which manifests itself when an officer takes on its usual role as a service provider (the officer trait) and the other which manifests itself when the officer plays the role of a citizen requiring a service (the citizen trait). We further assume that citizens cannot act as service providers under any circumstances. This leads to four distinct classes of officers which make up the officer sub-population at any point of time. Officers who do not demand bribes in their role as service providers and pay silently in their role as citizens (*O*_11_); officers who do not demand bribes in their role as service providers but register a complaint after paying a bribe in their role as citizens (*O*_12_); officers who demand a bribe in their role as service provides but pays a bribe silently in their role as citizens (*O*_21_); officers who demand a bribe in their role as service providers but registers a complaint after paying a briber in their role as citizens (*O*_22_). Such considerations allow us to partially symmetrize an asymmetric game [28].

A contentious issue in evolutionary game theory involves the specification of the update rule according to which the population is updated over successive generations. In the previous analysis, we used an update rule according to which a citizen (or officer) is selected to replace a randomly chosen citizen (or officer) in the next generation with a probability proportional to the difference in fitness between the two. This can be equivalently thought of as the individual being replaced imitating the strategy of the citizen (or officer) selected to replace her. In the finite population limit, this amounts to a local update imitation process [26,27].

In order to study the effect of changing the update rule on the equilibrium population structure, we analyse the partially symmetrized bribery game using the proportional imitation update rule [29,30] which has been used recently to study adversarial evolutionary games [31]. According to this rule, citizens and officers updates their strategies only when they incur a loss during a bribery interaction. Characterization of the loser depends on the strategies involved in the interaction and is determined by comparing the payoff an individual gets during an interaction to the payoff the same individual would have got had she used an alternative strategy. If the latter is larger, the individual changes its strategy with a probability proportional to the relative payoff of the new strategy. If the latter is smaller, the individual retains its original strategy. A citizen suffers a loss if the bribery incident goes unpunished while an officer suffers a loss if she does not demand a bribe or when she is prosecuted for taking a bribe. For example, if the two randomly selected individuals from the officer and citizen sub-populations are of type are *O*_22_ and *O*_11_ respectively, with the understanding that *O*_22_ plays the role of an officer who demands a bribe and *O*_11_ plays the role of a citizen who pays a bribe without complaining; the payoff that the officer and citizen would get as a result of that interaction is (*v* + *b*) and (*c* − *b*) respectively. The alternative strategy for the citizen to adopt against the officer who “demands a bribe” is to “pay but lodge a complaint”. Adopting such a strategy would result in a potential increase in payoff to (*c* − *b* − *t* + *kr*). Since by adopting the alternative strategy, the payoff for the citizen can potentially increase, the citizen switches its strategy from *O*_11_ to *O*_12_ with a probability

$$p_{O_{11}\to O_{12}}=\frac{c-b-t+kr}{2(c-b)-t+kr}$$ (14)

where the denominator corresponds to the sum of the payoffs to the citizen before and after the strategy switch.

The officer of type *O*_*22*_ retains its original strategy because employing the alternative strategy O_12_ would lead to a decrease in her payoff. The switching probabilities for all possible interactions between officers and citizens are given in S1 Table. The deterministic equations (see S1 Appendix for a detailed explanation of how these equations were obtained) for the time-evolution of the frequencies of the different strategies in the population can then be written as

$$\begin{array}{l} {\dot{O}}_{11}=\frac{\left(1-k)(c-b)O_{12}(O_{22}+O_{21}\right)}{2(c-b)-t}+\frac{kvO_{21}(O_{12}+O_{22}+C_{2})}{2v+b-p}-\frac{\left(c-b-t+kr)O_{11}(O_{21}+O_{22}\right)}{2(c-b)+kr-t}\\ \;-\frac{\left(v+b)O_{11}(O_{11}+O_{21}+C_{1}\right)}{2v+b}-\frac{\left(v+b-kp)O_{11}(O_{12}+O_{22}+C_{2}\right)}{2v+b-kp} \end{array}$$ (15)

$$\begin{array}{l} {\dot{O}}_{12}=\frac{\left(c-b-t+kr)O_{11}(O_{21}+O_{22}\right)}{2(c-b)+kr-t}+\frac{kvO_{22}(O_{12}+O_{22}+C_{2})}{2v+b-p}-\frac{\left(1-k)(c-b)O_{12}(O_{22}+O_{21}\right)}{2(c-b)-t}\\ \;-\frac{\left(v+b)O_{12}(O_{11}+O_{21}+C_{1}\right)}{2v+b}-\frac{\left(v+b-kp)O_{12}(O_{12}+O_{22}+C_{2}\right)}{2v+b-kp} \end{array}$$ (16)

$$\begin{array}{l} {\dot{O}}_{21}=\frac{\left(v+b)O_{11}(O_{11}+O_{21}+C_{1}\right)}{2v+b}+\frac{\left(v+b-kp)O_{11}(O_{12}+O_{22}+C_{2}\right)}{2v+b-kp}\\ \;+\frac{\left(1-k)(c-b)O_{22}(O_{22}+O_{21}\right)}{2(c-b)-t}-\frac{kvO_{21}(O_{12}+O_{22}+C_{2})}{2v+b-p}-\frac{\left(c-b-t+kr)O_{21}(O_{21}+O_{22}\right)}{2(c-b)+kr-t} \end{array}$$ (17)

$${\dot{O}}_{22}=-({\dot{O}}_{11}+{\dot{O}}_{12}+{\dot{O}}_{21})$$ (18)

$${\dot{C}}_{1}=\frac{\left(1-k)(c-b)C_{2}(O_{21}+O_{22}\right)}{2(c-b)-t}-\frac{\left(c-b-t+kr)C_{1}(O_{21}+O_{22}\right)}{2(c-b)-t+kr}$$ (19)

$${\dot{C}}_{1}=-{\dot{C}}_{2}$$ (20)

**Fig 10:**
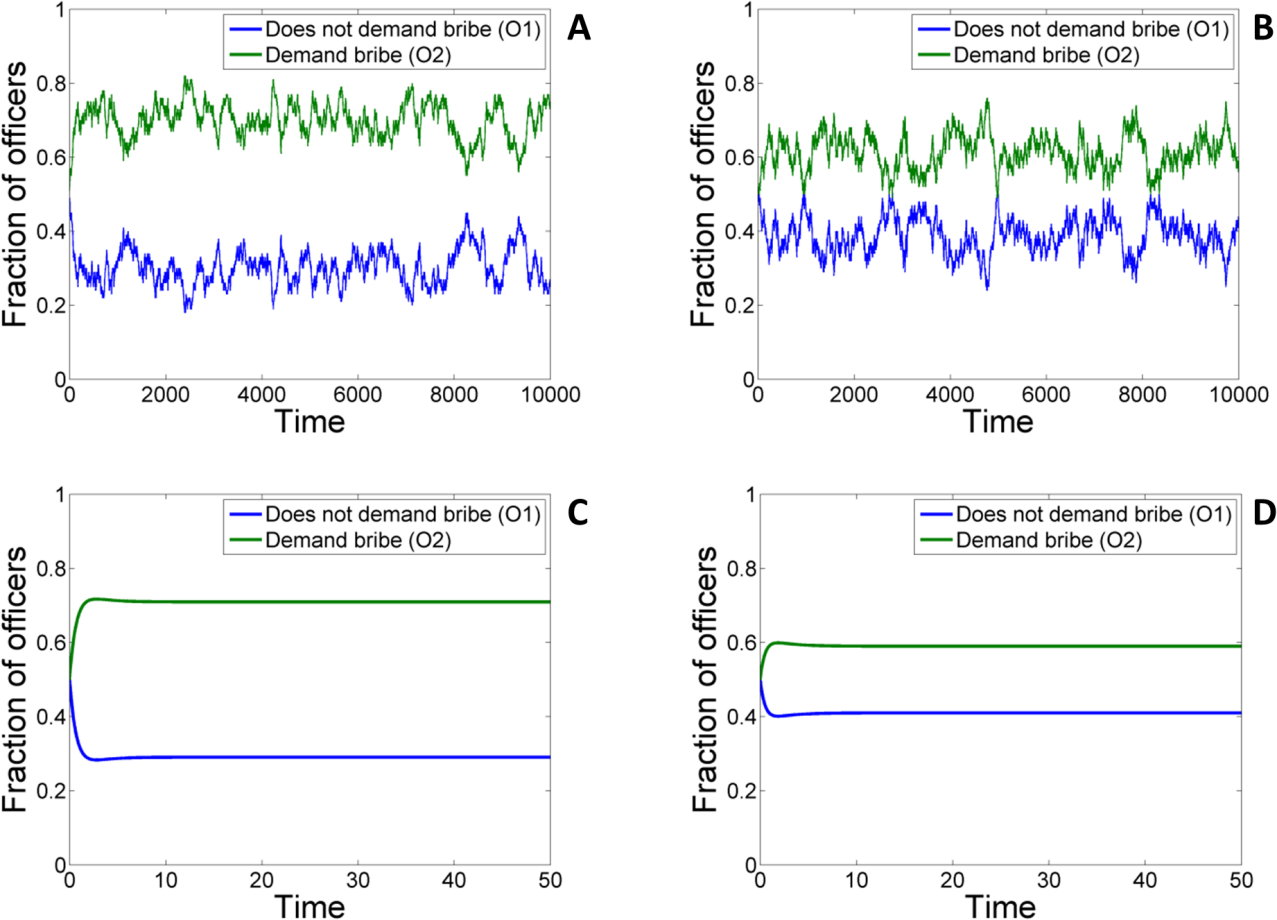
Time evolution of the total fraction of honest and corrupt officers in the population for stochastic ABS (A & B) and deterministic simulation (C & D) for p_*e*_ = 0 (A & C) and *p*_*e*_ = 0.5 (B & D). Other values of parameters: *c* = 1, *v* = 1, *p*_*o*_ = 1.3, *p*_*c*_ = 0, *k* = 0.4, *b* = 0.4, *r* = 0; *t* = 0.1. Number of officers in ABS: *N*_0_ = 100; Number of pure citizens in ABS: *N*_*C*_ = 100.

**Fig 11:**
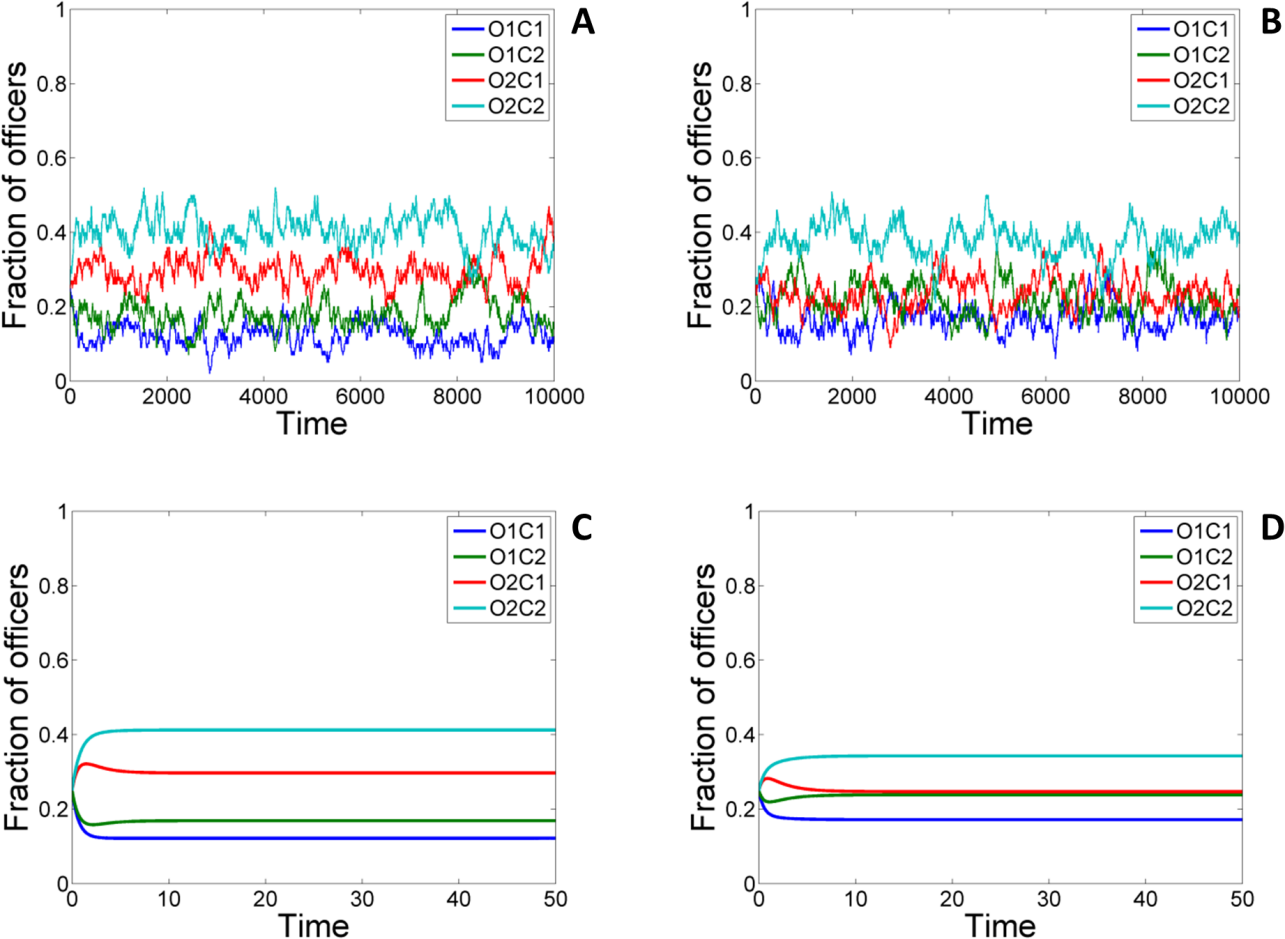
Time evolution of the frequencies of each of the different categories of officers officers in the partially symmetrized game. The panels show the outcome of the stochastic ABS (A & B) and deterministic simulations (C & D) for *p*_*e*_ = **0** (A & C) and *p*_*e*_ = **0.5** (B & D) case. Other values of parameters are: *c* = 1, *v* = 1, *p*_*o*_ = 1.3, *p*_*c*_ = 0, *k* = 0.4, *b* = 0.4, *r* = 0, *t* = 0.1. Number of officers in ABS: *N*_0_ = 100; Number of pure citizens in ABS: *N*_*C*_ = 100.

An agent based simulation (ABS) of the above model was also carried out to study the effects of stochasticity arising from finite population of citizens and officers. Figs 10 and 11 (A) and (C) shows the variation of frequency of the officers obtained from the deterministic model as well as the stochastic ABS model where the number of officers and citizens (including officers who can play the role of citizens) are 100 and 200 respectively. Even though substantial stochastic fluctuations about the mean value are observed, a comparison of the two figures in the large population limit (see S2 Fig and S3 Fig) shows that the equilibrium mean values of the frequencies obtained from the stochastic model converges to that obtained using the deterministic model described above.

We also used the deterministic equations to analyse the population structure of the different strategies when parameters like punishment, prosecution rate, bribe amount and cost of complaining are varied across a wide range of values in order to examine the conditions under which incidents of bribery can be reduced. Comparison with the results of imitation dynamics in the 4-strategy model is also helpful in understanding how update rules affect the outcome of the bribery game.

**Fig 12:**
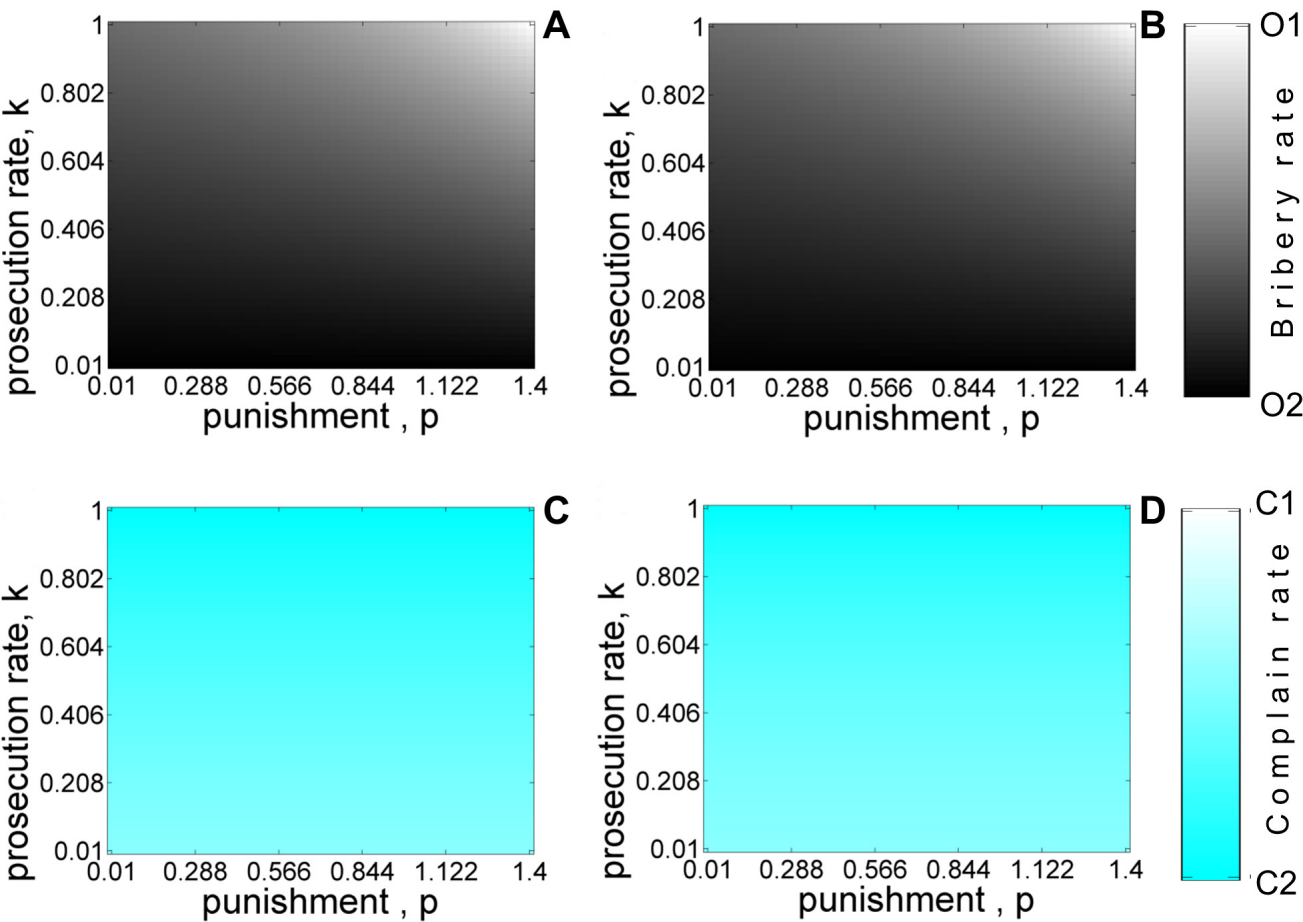
Equilibrium population for the alternative strategy exploration model as a function of punishment *p* and prosecution rate *k* with refund (A, C) and without-refund (B, D) for asymmetric liability scenario. Shades of white and black color denote the equilibrium abundance of *O*_1_ and *O*_2_ type of officers. Shades of white and cyan color denote the stationary frequencies of *C*_1_ and *C*_2_ type of citizens. The values of other parameters are: *c* = 1, *v* = 1, *b* = 0.4, *t* = 0.1. The initial condition corresponds to *x*_*C*1_ = 0.5, *x*_*C*2_ = 0.5, *x*_*O*1_ = 0.5, *x*_*O*2_ = 0.5

Fig 12 shows the phase diagram depicting how the evolution towards a society free of corruption depends on the prosecution rate and punishment with (Fig 12A and 12C) and without (Fig 12B and 12D) refunds. Such a society can be established only for very high prosecution rates and punishment in marked contrast to Fig 7. The equilibrium frequency of complaining citizens is independent of the punishment. Nevertheless, the asymmetric punishment rule does lead to a reduction in bribery for more reasonable values of punishment and prosecution rate as indicated by the coexistence of honest and corrupt officers (grey region of Fig 12A and 12B). The fixation of honest officers is correlated with the dominance of conscientious citizens who lodge a complaint. Increasing the initial number of honest officers and complaining citizens does not significantly alter the conditions under which incidents of harassment bribery are reduced.

Similar trends are observed when *p* is varied along with *b* and *t* respectively with coexistence of honest and corrupt officers observed over large regions of parameter space (see S4 Fig and S5 Fig). However, even in the coexistence phase, the population appears to be dominated by corrupt officers with honest officers making up a smaller fraction. These results suggest that even the asymmetric liability scenario is only marginally effective in reducing incidents of bribery in the alternate strategy model.

The partially symmetrized bribery game where officers possess two traits is quite useful in exploring the effects of empathy on bribery. The effects of empathy can be modelled by noting that a corrupt officer who is forced to pay a bribe when acting like a citizen (i.e. officers of type *O*_21_ and *O*_22_) can better empathize with the plight of the victimized citizens and will be less likely to demand bribes when they act as officers. The effect of empathy is manifest by changes in the officer’s trait from a corrupt to an honest officer. We assume that this transformation occurs with switching probability *p*_*e*_. When an individual of type *O*_21_ and *O*_22_ playing the role of a citizen (who either pays the bribe silently (*O*_21_) or pays and registers a complaint (*O*_22_)) interacts with a corrupt officer demanding a bribe (*O*_21_ or *O*_22_), three distinct transformations are possible. (i) The individual can change her citizen’s trait without empathizing i.e. *O*_21_ → *O*_22_ or *O*_22_ → *O*_21_. (ii) Does not change her citizen’s trait but show empathy for the victimized citizen by changing her officer’s trait i.e. *O*_21_ → *O*_11_ or *O*_22_ → *O*_12_. (iii) Changes both traits i.e. *O*_21_ → *O*_12_ or *O*_22_ → *O*_11_. See S2 Table for the new switching probabilities. These previously non-permissible transformations will lead to the appearance of new terms in the time-evolution equations for the frequencies. The modified set of deterministic equations in the presence of empathy is

$${\dot{O}}_{11}^{*}={\dot{O}}_{11}+p_{e}\left(1-\frac{c-b-t+kr}{2(c-b)-t+kr}\right)O_{21}(O_{21}+O_{22})+\frac{p_{e}(1-k)(c-b)}{2(c-b)-t}O_{22}(O_{21}+O_{22})$$ (21)

$${\dot{O}}_{12}^{*}={\dot{O}}_{12}+p_{e}\left(\frac{c-b-t+kr}{2(c-b)-t+kr}\right)O_{21}(O_{21}+O_{22})+p_{e}\left(1-\frac{\left(1-k)(c-b\right)}{2(c-b)-t}\right)O_{22}(O_{21}+O_{22})$$ (22)

$${\dot{O}}_{21}^{*}={\dot{O}}_{21}-p_{e}\left(1-\frac{c-b-t+kr}{2(c-b)-t+kr}\right)O_{21}(O_{21}+O_{22})+(1-p_{e})\left(\frac{\left(1-k)(c-b\right)}{2(c-b)-t}\right)O_{22}(O_{21}+O_{22})$$ (23)

$${\dot{O}}_{22}^{*}=-({\dot{O}}_{11}^{*}+{\dot{O}}_{12}^{*}+{\dot{O}}_{21}^{*})$$ (24)

$${\dot{C}}_{1}^{*}={\dot{C}}_{1}$$ (25)

$${\dot{C}}_{2}^{*}={\dot{C}}_{2}$$ (26)

Here ${\dot{O}}_{11},{\dot{O}}_{12},{\dot{O}}_{21},{\dot{O}}_{22},{\dot{C}}_{1},{\dot{C}}_{2}$ are given by Eqs (15)–(20) and the * denotes the modified time-derivatives of the frequencies when *p*_*e*_ ≠ 0.

Inclusion of empathy has a significant impact in reducing incidents of bribery as is evident from Figs 10 and 11 (see panels B, D) which shows the results of the deterministic (panels C,D) as well as the stochastic (panels A,B) ABS model without (panels A,C) and with (panels B, D) empathy.

## Discussion

The asymmetric penalty scenario proposed by Basu is successful in significantly reducing incidents of bribery only under certain restrictive conditions. The effect of asymmetry in punishment on the extent of reduction in bribery incidents depends on the manner in which the population of citizens and officers are updated every generation. When the selection procedure is inspired by biological evolution, complete eradication of bribery is possible under certain circumstances. Such an update rule is akin to players with lower payoff imitating the strategy of players who received a higher payoff, in the next round of the game. In such scenarios, significant reduction in bribery is observed if refunds are allowed. Creation of a platform where citizens can easily report about the bribery demands can also facilitate significant reduction in bribery provided a sufficient number of people report such incidents. This is because such reporting can lead to potential shaming of corrupt public officials and consequent reduction in their social capital thereby discouraging them from taking bribes even in the absence of any compensation for the bribe-giver.

The incentive for refund in the *asymmetric liability* scenario leads to a situation where a demand for too low or too high a bribe amount leads to the elimination of corrupt officers (O2) from the population. They survive only if the bribe amount is optimised to some moderate range. This suggests that unless the citizens perceive the bribe demand to be a fair price for the service sought, corrupt officers cannot be sustained in the population. This result also hints towards a possible connection of our game with the ultimatum game [32] where fairness wins over rational choices as an evolutionarily stable strategy. It can be shown that the bribery game reduces to the ultimatum game for special values of parameters (*t = 0*, *k = 0* and *v = 0*). However, it is important to note that the reason for the emergence of fairness in the bribery game is due to the incentive for refund and asymmetric penalty unlike the situation in the ultimatum game where the dominance of fair proposers arises because they possess information about the past encounters with the responder [32].

When the mode of selection is based on exploration of an alternative strategy with a player updating her strategy with a probability proportional to the payoff of the winner’s strategy, only if she loses in the last round of the game, reduction in incidents of bribery is less pronounced and bribery is never eliminated. Refunding the bribe amount does not appear to significantly alter the population structure in this scenario. Honest officers are tempted to increase their payoff by resorting to corrupt practices of fellow officers. This leads to a constant generation of corrupt officers from the pool of honest officers. As a consequence, the fraction of corrupt offices in the population remains quite high even when the penalty for bribe taking is large. The latter update rule seems more appropriate in the context of decision-making in situations of social conflict manifest in bribery dynamics. Empathetic officers who decide to switch to an honest strategy after facing bribe demands in their role as citizens can also reduce the incidents of bribery. The extent of reduction depends on the level of empathy that was quantified through the switching probability.

Tackling the scourge of bribery is a difficult and complex task and our analysis suggests that no single policy prescription can be successful in eradicating it. It would perhaps be more pragmatic to look at a combination of technological fixes and public policies targeting the myriad underlying causes of bribery in order to effect reduction in bribery and ease the toll it takes on public finances.

## Materials and Methods

Deterministic solution: The replicator equation for the five-strategy and four-strategy model and deterministic equations for the alternative strategy model was solved using Runge-Kutta (RK4) algorithm. We start with a fixed initial condition when all the strategies are equally abundant in the population. The equations are numerically solved until equilibrium is established.

Agent Based Simulation (ABS): Stochastic ABS was carried out for the alternative strategy model using two different population sizes. The population of the group which consists of citizens only (*C*_1_, *C*_2_) was taken to be 100. Since officers can also act as citizens (*O*_11_, *O*_12_, *O*_21_, *O*_22_), the total population of such officers and citizens who have dual traits was also taken to be 100. At each time step a random officer and citizen is picked up from the pool of officers (*O*_11_, *O*_12_, *O*_21_, *O*_22_) and citizens (*O*_11_, *O*_12_, *O*_21_, *O*_22_, *C*_1_, *C*_2_). A loser from each category is determined by comparing the payoff to the officer (and citizen) with the payoff she would have got with the alternative strategy. The loser in the interaction then changes her strategy to the alternative option with a probability proportional to the payoff of alternative strategy. The population is evolved till the equilibrium population structure is established. All simulations were carried out using the Matlab package.

Simulations to show how the equilibrium population structure changes when the initial fraction of different strategies represented in the population are varied (Fig 9) were carried out using the Mathematica package.

## Supporting Information

S1 Appendix Detailed derivation of Eq (15). (DOCX)

S1 Fig Game tree for the four-strategy harassment bribery game. (TIF)

S2 Fig Time evolution of the total fraction of honest and corrupt officers in the population for stochastic ABS (A & B) and deterministic simulation (C & D) for *p_e_* = 0 (A & C) and *p*_*e*_ = 0.5 (B & D). Other values of parameters: *c* = 1, *v* = 1, *p*_*o*_ = 1.3, *p*_*c*_ = 0, *k* = 0.4, *b* = 0.4, *r* = 0, *t* = 0.1. Number of officers in ABS: *N*_*O*_ = 2000; Number of pure citizens in ABS: *N*_*C*_ = 2000.
(TIF)

S3 Fig Time evolution of the frequencies of different strategies adopted by officers in the partially symmetrized game for stochastic ABS (A & B) and deterministic simulations (C & D) for *p*_*e*_ = 0 (A & C) and *p*_*e*_ = 0.5 (B & D) case. Other values of parameters are: *c* = 1, *v* = 1, *p*_*o*_ = 1.3, *p*_*c*_ = 0, *k* = 0.4, *b* = 0.4, *r* = 0, *t* = 0.1. Number of officers in ABS: *N*_*O*_ = 2000; Number of pure citizens in ABS: *N*_*C*_ = 2000.
(TIF)

S4 Fig Equilibrium population structure for the alternative strategy exploration model as a function of bribe amount (*b*), and punishment (*p*) with refund (A, C) and without-refund (B, D) for asymmetric liability scenario. Shades of white and black color denote the equilibrium abundance of *O*_1_ and *O*_2_ type of officers. Shades of white and cyan color denote the stationary frequencies of *C*_1_ and *C*_2_ type of citizens. The values of other parameters are: *c* = 1, *v* = 1, *k* = 0.6, *t* = 0.1. The initial condition corresponds to *x*_*C*1_ = 0.5, *x*_*C*2_ = 0.5, *x*_*O*1_ = 0.5, *x*_*O*2_ = 0.5.
(TIF)

S5 Fig Equilibrium population structure for the alternative strategy exploration model as a function of punishment (*p*) and cost of complaining (*t*) with refund (A, C) and without-refund (B, D) for asymmetric liability scenario. Shades of white and black color denote the equilibrium abundance of *O*_1_ and *O*_2_ type of officers. Shades of white and cyan color denote the stationary frequencies of *C*_1_ and *C*_2_ categories of citizens. The values of other parameters are: *c* = 1, *v* = 1, *k* = 0.6, *b* = 0.4 The initial condition corresponds to *x*_*C*1_ = 0.5, *x*_*C*2_ = 0.5, *x*_*O*1_ = 0.5, *x*_*O*2_ = 0.5.
(TIF)

S1 Table The switching probabilities for all possible interactions between officers and citizens for the “alternative strategy exploration model”. (DOCX)

S2 Table The additional/modified terms of switching probabilities of possible interactions in the “alternative strategy exploration model” with empathy. (DOCX)
