# Supplementary material for: Bribe and Punishment: An Evolutionary Game-Theoretic Analysis of Bribery
Source: PLoS One. 2015 Jul 23;10(7):e0133441. doi: 10.1371/journal.pone.0133441 (PMC4512696; doi:10.1371/journal.pone.0133441)
Supplement: S1 Appendix — (DOCX) [file pone.0133441.s001.docx]

**S1 Appendix**: Detailed derivation of Equation (15)

The transition matrix $T_{1}$ for the alternative strategy exploration model of partially symmetrized game (without empathy term) for officers is obtained using information given in Table S1. Each element of the matrix represents the probability of transition from rows’ strategy into columns’ strategy. For example the probability of transition from strategy $O_{21}$ to $O_{11}$ is $\frac{kvO_{21}(O_{12}+O_{22}+C_{2})}{2v+b-p}$ represented by the matrix element $T_{1}(O_{21},O_{11})$ or $T_{1}(3,1)$. This can be easily verified by looking at rows 19, 20 and 21 of Table S1.

The rate of change of frequency of a strategy is the sum total of transitions into the given strategy minus the sum total of transitions of the given strategy into others strategies. For example, for the case of strategy$O_{11}$, the rate of change of frequency is

|  |  |  |
| --- | --- | --- |

Here$j\in\left\{O_{11},O_{12},O_{21},O_{22}\right\}$. The above equation implies that the rate of change of frequency of the strategy may be obtained by summing up terms of column of the transition matrix $T_{1}$ and subtracting the row terms for a given strategy. For $O_{11}$ it is given by

|  |
| --- |

This leads to equation (15) given in the main text. Similarly, we can obtain other equations (16) – (20) using the same methodology. Equation (18) and (20) follows from the fact that the population size of officers and pure citizens are conserved separately i.e.

| $\begin{matrix} O_{11}+O_{12}+O_{21}+O_{22}=1 \\ C_{1}+C_{2}=1 \end{matrix}$ |
| --- |

Equations (21)-(26) can be derived using Table S2 by following the same method as outlined above.
