# Supplementary figures and images for: Bribe and Punishment: An Evolutionary Game-Theoretic Analysis of Bribery
Source: PLoS One. 2015 Jul 23;10(7):e0133441. doi: 10.1371/journal.pone.0133441 (PMC4512696; doi:10.1371/journal.pone.0133441)

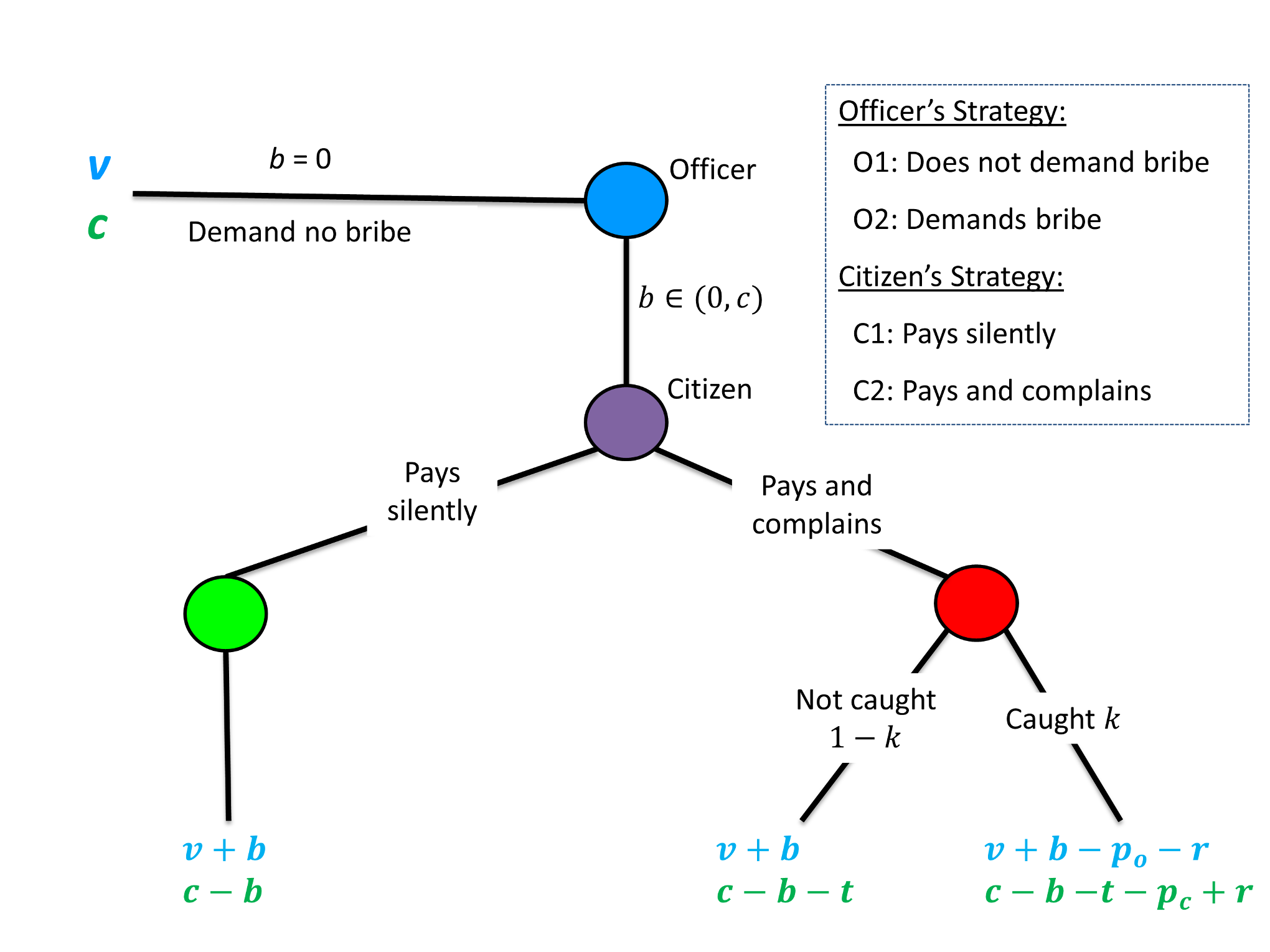

Supplement: S1 Fig — (TIF) [file pone.0133441.s002.tif]

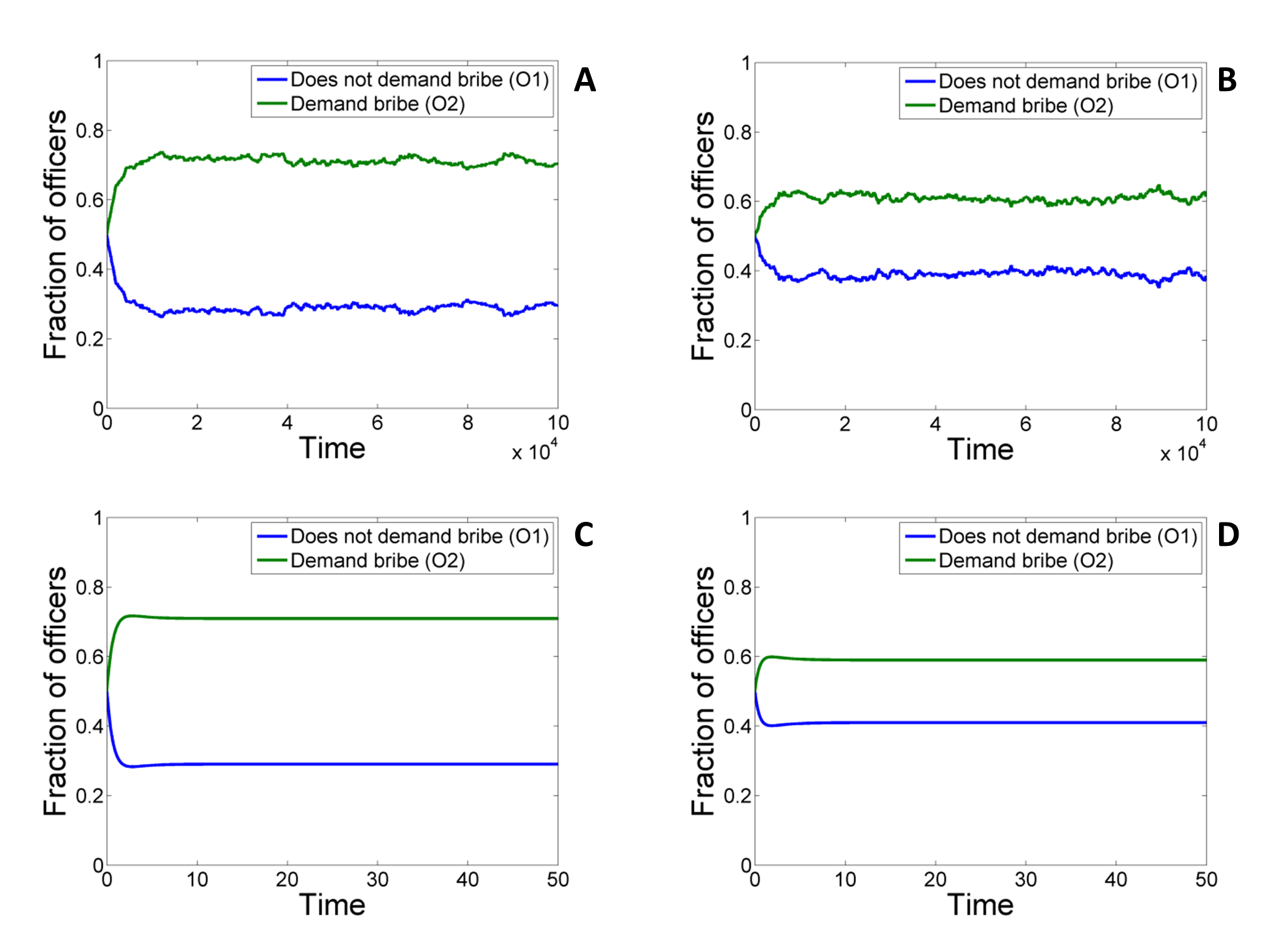

Supplement: S2 Fig — Other values of parameters: c = 1, v = 1, p o = 1.3, p c = 0, k = 0.4, b = 0.4, r = 0, t = 0.1. Number of officers in ABS: N O = 2000; Number of pure citizens in ABS: N C = 2000. (TIF) [file pone.0133441.s003.tif]

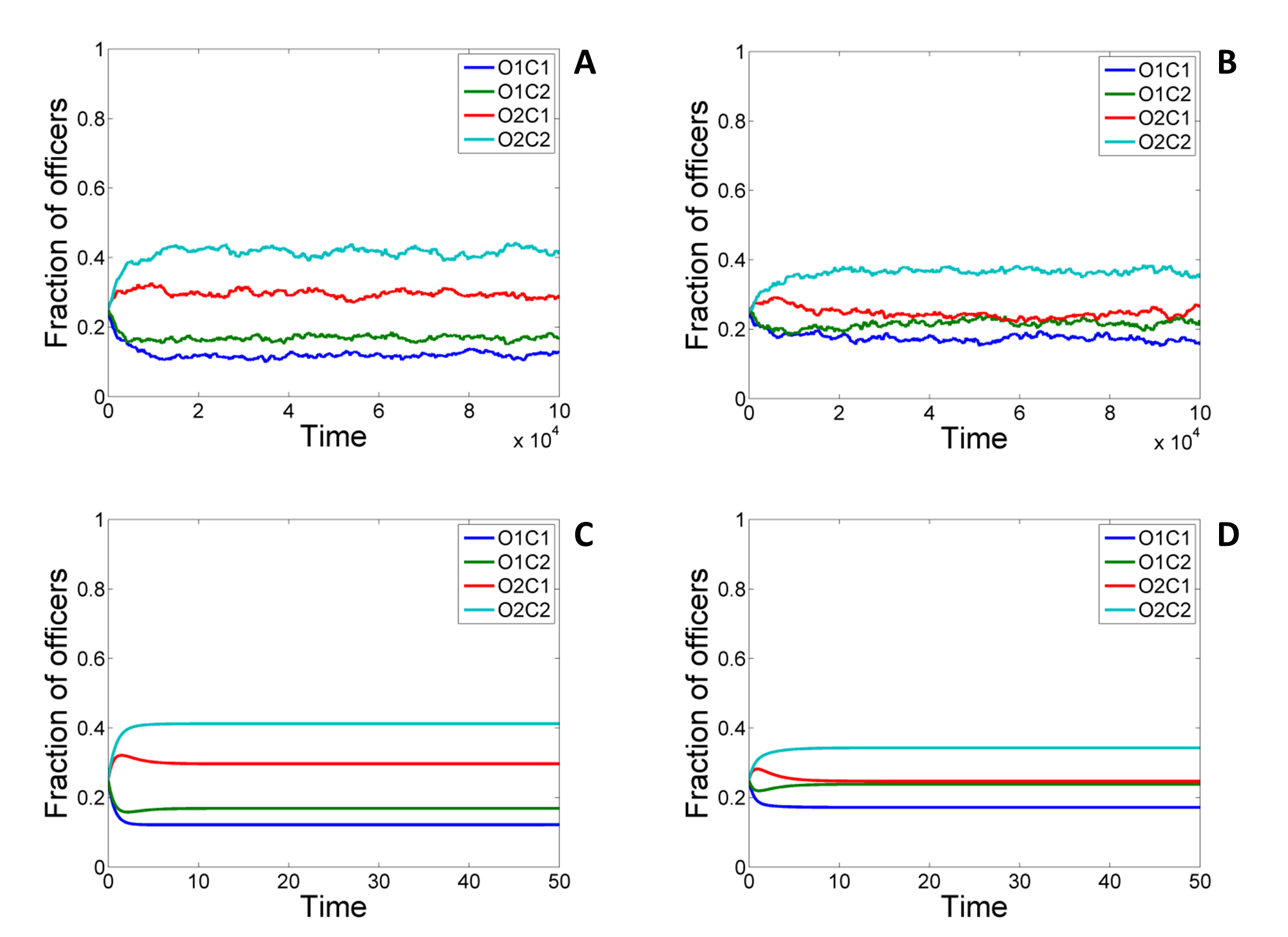

Supplement: S3 Fig — Other values of parameters are: c = 1, v = 1, p o = 1.3, p c = 0, k = 0.4, b = 0.4, r = 0, t = 0.1. Number of officers in ABS: N O = 2000; Number of pure citizens in ABS: N C = 2000. (TIF) [file pone.0133441.s004.tif]

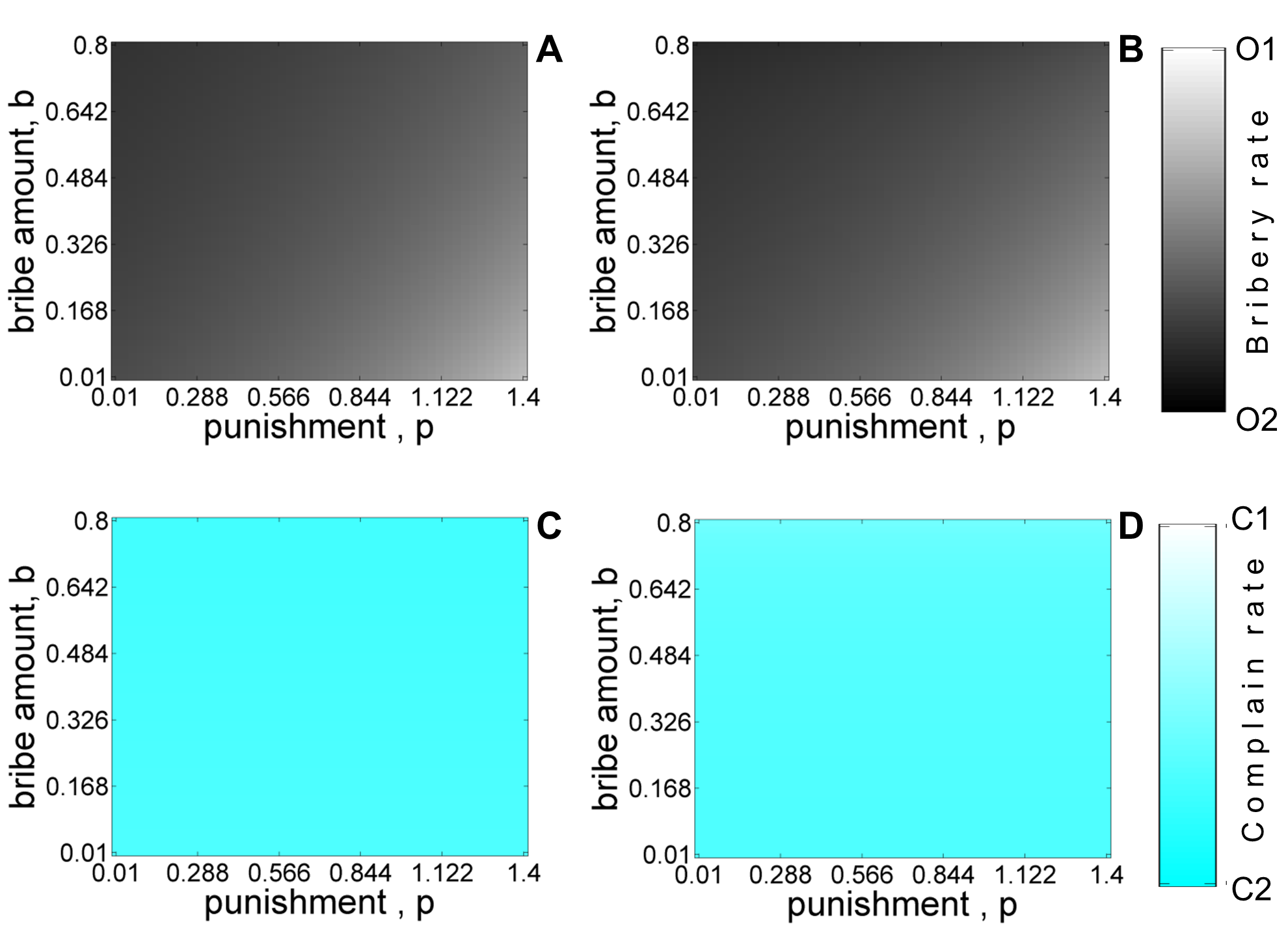

Supplement: S4 Fig — Shades of white and black color denote the equilibrium abundance of O 1 and O 2 type of officers. Shades of white and cyan color denote the stationary frequencies of C 1 and C 2 type of citizens. The values of other parameters are: c = 1, v = 1, k = 0.6, t = 0.1. The initial condition corresponds to x C1 = 0.5, x C2 = 0.5, x O1 = 0.5, x O2 = 0.5. (TIF) [file pone.0133441.s005.TIF]

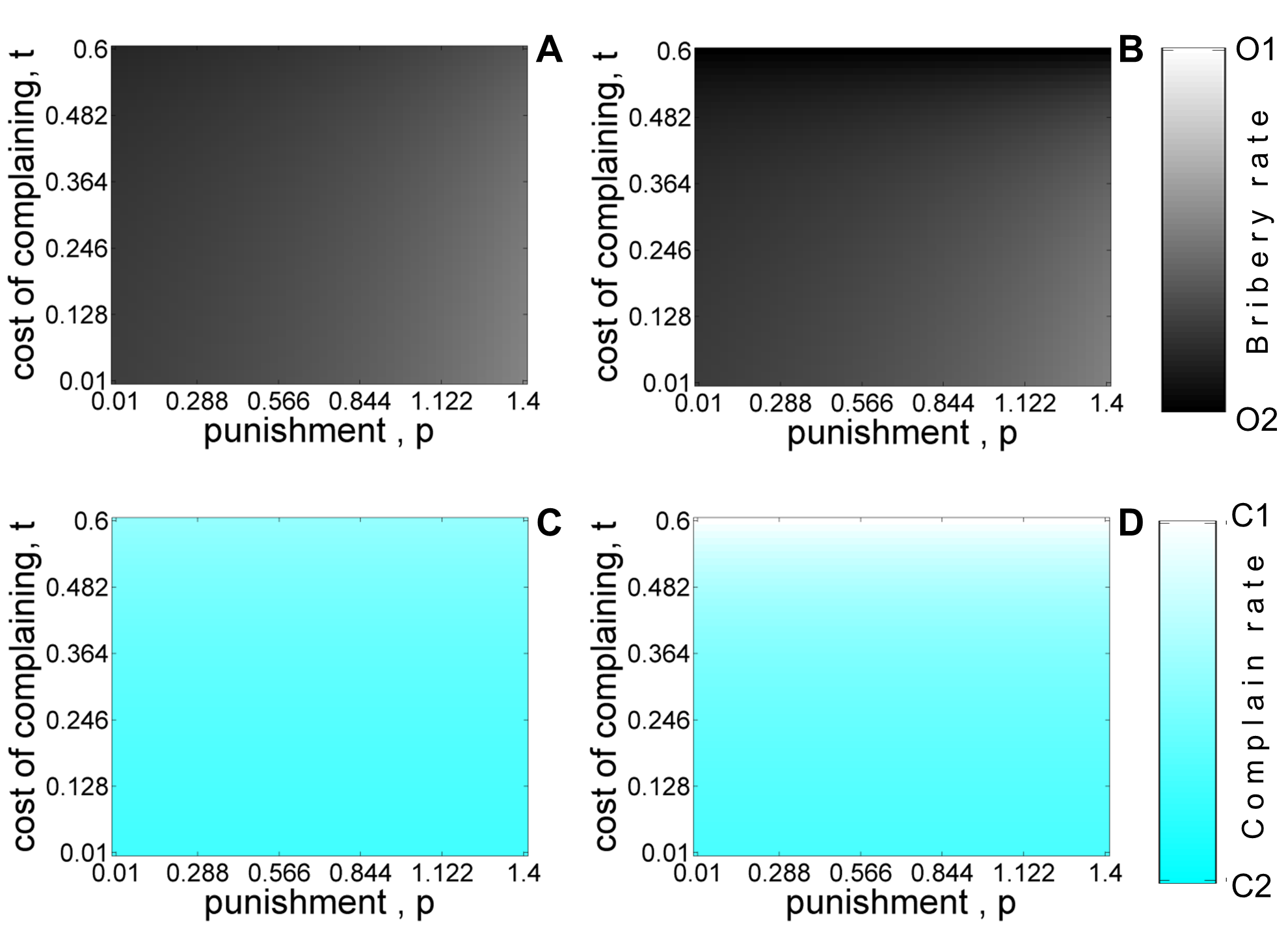

Supplement: S5 Fig — Shades of white and black color denote the equilibrium abundance of O 1 and O 2 type of officers. Shades of white and cyan color denote the stationary frequencies of C 1 and C 2 categories of citizens. The values of other parameters are: c = 1, v = 1, k = 0.6, b = 0.4 The initial condition corresponds to x C1 = 0.5, x C2 = 0.5, x O1 = 0.5, x O2 = 0.5. (TIF) [file pone.0133441.s006.TIF]
