# Supplementary material for: Bribe and Punishment: An Evolutionary Game-Theoretic Analysis of Bribery
Source: PLoS One. 2015 Jul 23;10(7):e0133441. doi: 10.1371/journal.pone.0133441 (PMC4512696; doi:10.1371/journal.pone.0133441)
Supplement: S1 Table — (DOCX) [file pone.0133441.s007.docx]

**S1Table**: The switching probabilities for all possible interactions between officers and citizens for the “alternative strategy exploration model”

| S.no. | Officer | Citizen | Payoff of officer | Payoff of citizen | Transition | Switching probability |
| --- | --- | --- | --- | --- | --- | --- |
| 1 | O_11_ (O_1_) | O_11_ (C_1_) | v | c | O_11_ → O_21_ | (v+b)/(2v+b) |
| 2 | O_11_ (O_1_) | O_12_ (C_2_) | v | c | O_11_ → O_21_ | (v+b−kp)/(2v+b−kp) |
| 3 | O_11_ (O_1_) | O_21_ (C_1_) | v | c | O_11_ → O_21_ | (v+b)/(2v+b) |
| 4 | O_11_ (O_1_) | O_22_ (C_2_) | v | c | O_11_ → O_21_ | (v+b−kp)/(2v+b−kp) |
| 5 | O_11_ (O_1_) | C_1_ | v | c | O_11_ → O_21_ | (v+b)/(2v+b) |
| 6 | O_11_ (O_1_) | C_2_ | v | c | O_11_ → O_21_ | (v+b−kp)/(2v+b−kp) |
| 7 | O_12_ (O_1_) | O_11_ (C_1_) | v | c | O_12_ → O_22_ | (v+b)/(2v+b) |
| 8 | O_12_ (O_1_) | O_12_ (C_2_) | v | c | O_12_ → O_22_ | (v+b−kp)/(2v+b−kp) |
| 9 | O_12_ (O_1_) | O_21_ (C_1_) | v | c | O_12_ → O_22_ | (v+b)/(2v+b) |
| 10 | O_12_ (O_1_) | O_22_ (C_2_) | v | c | O_12_ → O_22_ | (v+b−kp)/(2v+b−kp) |
| 11 | O_12_ (O_1_) | C_1_ | v | c | O_12_ → O_22_ | (v+b)/(2v+b) |
| 12 | O_12_ (O_1_) | C_2_ | v | c | O_12_ → O_22_ | (v+b−kp)/(2v+b−kp) |
| 13 | O_21_ (O_2_) | O_11_ (C_1_) | v+b | c−b | O_11_ → O_12_ | (c−b−t+kr)/(2c−2b−t+kr) |
| 14 | O_21_ (O_2_) | O_21_ (C_1_) | v+b | c−b | O_21_ → O_22_ | (c−b−t+kr)/(2c−2b−t+kr) |
| 15 | O_21_ (O_2_) | C_1_ | v+b | c−b | C_1_ → C_2_ | (c−b−t+kr)/(2c−2b−t+kr) |
| Not prosecuted | | | | | | |
| 16 | O_21_ (O_2_) | O_12_ (C_2_) | v+b | c−b−t | O_12_ → O_11_ | (1−k)(c−b)/(2c−2b−t) |
| 17 | O_21_ (O_2_) | O_22_ (C_2_) | v+b | c−b−t | O_22_ → O_21_ | (1−k)(c−b)/(2c−2b−t) |
| 18 | O_21_ (O_2_) | C_2_ | v+b | c−b−t | C_2_ → C_1_ | (1−k)(c−b)/(2c−2b−t) |
| Prosecuted | | | | | | |
| 19 | O_21_ (O_2_) | O_12_ (C_2_) | v+b−p | c−b−t+r | O_21_ → O_11_ | kv/(2v+b−p) |
| 20 | O_21_ (O_2_) | O_22_ (C_2_) | v+b−p | c−b−t+r | O_21_ → O_11_ | kv/(2v+b−p) |
| 21 | O_21_ (O_2_) | C_2_ | v+b−p | c−b−t+r | O_21_ → O_11_ | kv/(2v+b−p) |
|  | | | | | | |
| 22 | O_22_ (O_2_) | O_11_ (C_1_) | v+b | c−b | O_11_ → O_12_ | (c−b−t+kr)/(2c−2b−t+kr) |
| 23 | O_22_ (O_2_) | O_21_ (C_1_) | v+b | c−b | O_21_ → O_22_ | (c−b−t+kr)/(2c−2b−t+kr) |
| 24 | O_22_ (O_2_) | C_1_ | v+b | c−b | C_1_ → C_2_ | (c−b−t+kr)/(2c−2b−t+kr) |
| Not prosecuted | | | | | | |
| 25 | O_22_ (O_2_) | O_12_ (C_2_) | v+b | c−b−t | O_12_ → O_11_ | (1−k)(c−b)/(2c−2b−t) |
| 26 | O_22_ (O_2_) | O_22_ (C_2_) | v+b | c−b−t | O_22_ → O_21_ | (1−k)(c−b)/(2c−2b−t) |
| 27 | O_22_ (O_2_) | C_2_ | v+b | c−b−t | C_2_ → C_1_ | (1−k)(c−b)/(2c−2b−t) |
| Prosecuted | | | | | | |
| 28 | O_22_ (O_2_) | O_12_ (C_2_) | v+b−p | c−b−t+r | O_22_ → O_12_ | kv/(2v+b−p) |
| 29 | O_22_ (O_2_) | O_22_ (C_2_) | v+b−p | c−b−t+r | O_22_ → O_12_ | kv/(2v+b−p) |
| 30 | O_22_ (O_2_) | C_2_ | v+b−p | c−b−t+r | O_22_ → O_12_ | kv/(2v+b−p) |
