# Supplementary material for: Bribe and Punishment: An Evolutionary Game-Theoretic Analysis of Bribery
Source: PLoS One. 2015 Jul 23;10(7):e0133441. doi: 10.1371/journal.pone.0133441 (PMC4512696; doi:10.1371/journal.pone.0133441)
Supplement: S2 Table — (DOCX) [file pone.0133441.s008.docx]

**S2 Table**: The additional/modified terms of switching probabilities of possible interactions in the “alternative strategy exploration model” with empathy

| S.no. | Officer | Citizen | Payoff of officer | Payoff of citizen | Transition | Switching probability |
| --- | --- | --- | --- | --- | --- | --- |
| Not prosecuted | | | | | | |
| 1 | O_21_ (O_2_) | O_22_ (C_2_) | v+b | c−b−t | O_22_ → O_21_ | (1−p_e_)(1−k)(c−b) /(2c−2b−t) |
| 2 | O_21_ (O_2_) | O_22_ (C_2_) | v+b | c−b−t | O_22_ → O_11_ | p_e_(1−k)(c−b)/(2c−2b−t) |
| 3 | O_21_ (O_2_) | O_22_ (C_2_) | v+b | c−b−t | O_22_ → O_12_ | p_e_ [1 − (1−k)(c−b) /(2c−2b−t)] |
| 4 | O_22_ (O_2_) | O_22_ (C_2_) | v+b | c−b−t | O_22_ → O_21_ | (1−p_e_)(1−k)(c−b) /(2c−2b−t) |
| 5 | O_22_ (O_2_) | O_22_ (C_2_) | v+b | c−b−t | O_22_ → O_11_ | p_e_(1−k)(c−b)/(2c−2b−t) |
| 6 | O_22_ (O_2_) | O_22_ (C_2_) | v+b | c−b−t | O_22_ → O_12_ | p_e_ [1 − (1−k)(c−b) /(2c−2b−t)] |
|  | | | | | | |
| 7 | O_21_ (O_2_) | O_21_ (C_1_) | v+b | c−b | O_21_ → O_22_ | (1−p_e_) (c−b−t+kr) /(2c−2b−t+kr) |
| 8 | O_21_ (O_2_) | O_21_ (C_1_) | v+b | c−b | O_21_ → O_12_ | p_e_ (c−b−t+kr) /(2c−2b−t+kr) |
| 9 | O_21_ (O_2_) | O_21_ (C_1_) | v+b | c−b | O_21_ → O_11_ | p_e_ [1−(c−b−t+kr) /(2c−2b−t+kr)] |
| 10 | O_22_ (O_2_) | O_21_ (C_1_) | v+b | c−b | O_21_ → O_22_ | (1−p_e_) (c−b−t+kr) /(2c−2b−t+kr) |
| 11 | O_22_ (O_2_) | O_21_ (C_1_) | v+b | c−b | O_21_ → O_12_ | p_e_ (c−b−t+kr) /(2c−2b−t+kr) |
| 12 | O_22_ (O_2_) | O_21_ (C_1_) | v+b | c−b | O_21_ → O_11_ | p_e_ [1− (c−b−t+kr) /(2c−2b−t+kr)] |
